# Magnetically Induced Catalytic Reduction of Biomass-Derived Oxygenated Compounds in Water

**DOI:** 10.1021/acscatal.2c01696

**Published:** 2022-07-01

**Authors:** Christian Cerezo-Navarrete, Irene Mustieles Marin, Héctor García-Miquel, Avelino Corma, Bruno Chaudret, Luis M. Martínez-Prieto

**Affiliations:** † Instituto de Tecnología Química, Universitat Politècnica de València (UPV), Avenida de los Naranjos S/N, 46022 Valencia, Spain; ‡ LPCNO, Laboratoire de Physique et Chimie des Nano-Objets, INSA, CNRS, UPS, Université de Toulouse, 135 Avenue de Rangueil, F-31077 Toulouse, France; § ITEAM Research Institute, Universitat Politécnica de Valencia, Camino de Vera s/n, 46022 Valencia, Spain; ∥ Departamento de Química Inorgánica (University of Seville), Instituto de Investigaciones Químicas (CSIC-US); Avenida Americo Vespucio 49, 41092 Seville, Spain

**Keywords:** magnetic nanoparticles, core−shell nanoparticles, magnetically induced catalysis, hydrogenation reactions, biomass-derived compounds

## Abstract

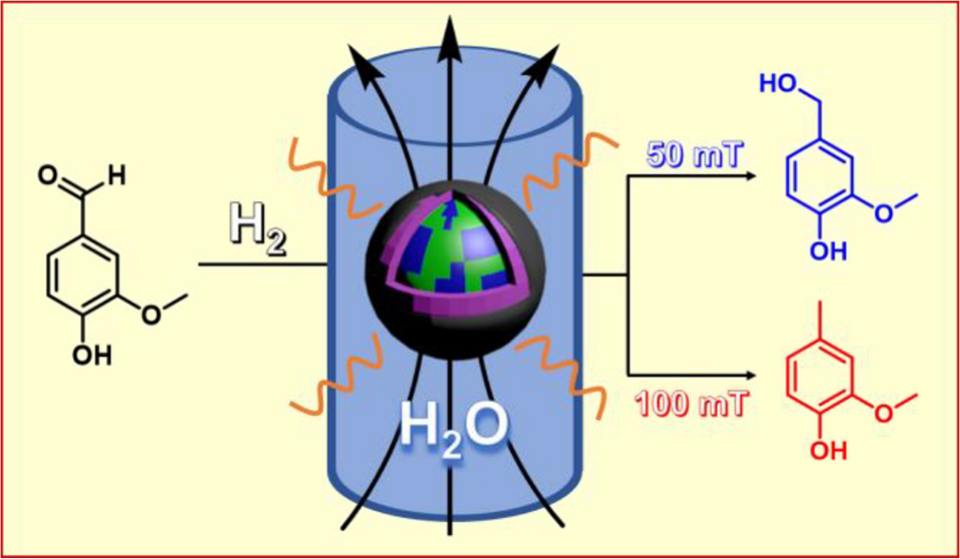

The development of energetically efficient processes for the aqueous reduction of biomass-derived compounds into chemicals is key for the optimal transformation of biomass. Herein we report an early example of the reduction of biomass-derived oxygenated compounds in water by magnetically induced catalysis. Non-coated and carbon-coated core–shell **FeCo@Ni** magnetic nanoparticles were used as the heating agent and the catalyst simultaneously. In this way it was possible to control the product distribution by adjusting the field amplitude applied during the magnetic catalysis, opening a precedent for this type of catalysis. Finally, the encapsulation of the magnetic nanoparticles in carbon (**FeCo@Ni@C**) strongly improved the stability of the magnetic catalyst in solution, making its reuse possible up to at least eight times in dioxane and four times in water.

## Introduction

Magnetic induction heating is an emerging subject in the field of catalysis that represents a promising alternative to conventional heating.^1^ It is based on the use of hysteresis losses produced in ferromagnetic materials by the presence of an alternating magnetic field (AMF). The use of magnetic nanoparticles (MagNPs) to transform electromagnetic energy into heat presents numerous advantages, among which the most important are (i) the short warming time, which allows catalytic process to be rapidly started or stopped, and (ii) the much more efficient energy transfer, since heating comes from inside the catalyst itself. All this makes magnetic heating an attractive technology for performing catalytic reactions in both solution^2−4^ and the gas phase.^5−11^ In particular, through magnetic catalysis in solution, it is possible to carry out reactions that normally requires high temperatures and pressures under milder conditions.^12−15^ This is due to the generation of a local high temperature at the catalyst surface under an AMF, which produces “hot spots” at temperatures much higher than the boiling point of the solvent, creating a vapor layer around the MagNPs and allowing transformations at lower pressures. Recently, the surface temperature (*T*_surf_) of magnetically heated MagNPs in solution was determined using a catalytic approach.^16^*T*_surf_ being high above the bulk temperature of the solution (*T*_bulk_) explains the exceptional catalytic performances of MagNPs in solution by magnetic heating. However, at high magnetic fields the activity of these MagNPs decreases due to sintering,^8,13,16^ which reduces the catalytic properties of the magnetic nanocatalysts and thus their lifetime. In this regard, MagNPs encapsulated in carbon have demonstrated to be efficient sinter-resistant heating agents in heterogeneous catalysis thanks to their higher stability, which avoids the agglomeration of the MagNPs under magnetic excitation.^17^

Hydrogenation and hydrodeoxygenation (HDO) are reactions involved in the transformation of biomass-derived oxygenated compounds.^18−21^ Hydrogenation, where molecular H_2_ is used to reduce unsaturated compounds, is one of the most important reactions for the formation of fine chemicals.^22−24^ On the other hand, the HDO of carbonyl groups is attracting a lot of attention in biomass valorization, since it directly produces the corresponding alkyl compounds.^25−28^ While HDO reactions are mainly carried out at high temperatures and pressures, hydrogenation reactions normally require milder conditions. However, accessing an active catalyst that simultaneously operates in hydrogenation and HDO processes is still a great challenge. Recently, Leitner et al., were able to control the formation of the hydrogenated or hydrodeoxygenated products using the same Rh catalyst by changing the reaction temperature.^29^ Similar temperature-controlled selectivity in the water-phase conversion of the biomass platform molecule vanillin was reported by Wang et al. in 2018.^30^ Both magnetically induced hydrogenation and HDO reactions were recently exploited for the transformation of biomass to value-added chemicals in solution. In 2019, Asensio et al. reported that colloidal iron carbide nanoparticles decorated with ruthenium (Fe_2.2_C@Ru) effectively performed the selective HDO of biomass platform molecules under mild conditions.^12^ The same authors also described bimetallic FeNi NPs (FeNi_3_@Ni) for the HDO of lignocellulose-derived products using magnetic induction.^13^ On the other hand, Gyergyek et al. recently reported a couple of heterogeneous magnetically heated catalysts for transforming biomass-derived compounds in solution.^14,15^ However, the main limitation of these works is the use of organic solvents as reaction media, while most biomass-derived compounds are only soluble in water.

The production of fine chemicals and fuels via the catalytic transformation of biomass is an attractive alternative to the use of fossil resources.^31−33^ However, since most catalysts are immiscible or incompatible with water, many of these catalytic reactions are carried out in organic solvents despite many biomass derivatives being soluble exclusively in aqueous media.^34^ Indeed, real biomass products are mostly soluble in water, which is often claimed to be the greenest solvent due to its nontoxicity and environmental compatibility. It is thus of interest to design robust catalytic systems for the transformation of biomass into chemicals that are able to operate in aqueous media and avoid extra separations, in some cases, and the use of organic solvents.

Herein, we present core–shell **FeCo@Ni** MagNPs for magnetically induced catalysis in solution, where the FeCo core (heating source) produces heat in close proximity to the Ni shell (catalytically active metal). In this work, we observed for the first time a magnetic-field-controlled selectivity of hydrogenation and HDO in the reduction of a biomass-derived molecule, namely 5-hydroxymethylfurfural (HMF). In addition, after the encapsulation of **FeCo@Ni** in carbon (**FeCo@Ni@C**), the MagNPs became highly active, selective, and stable in the aqueous reduction of biomass-derived oxygenated compounds by magnetic heating, representing major progress in energetically efficient catalytic transformations of biomass.

## Results and Discussion

### Synthesis and Characterization

**Figure 1:**
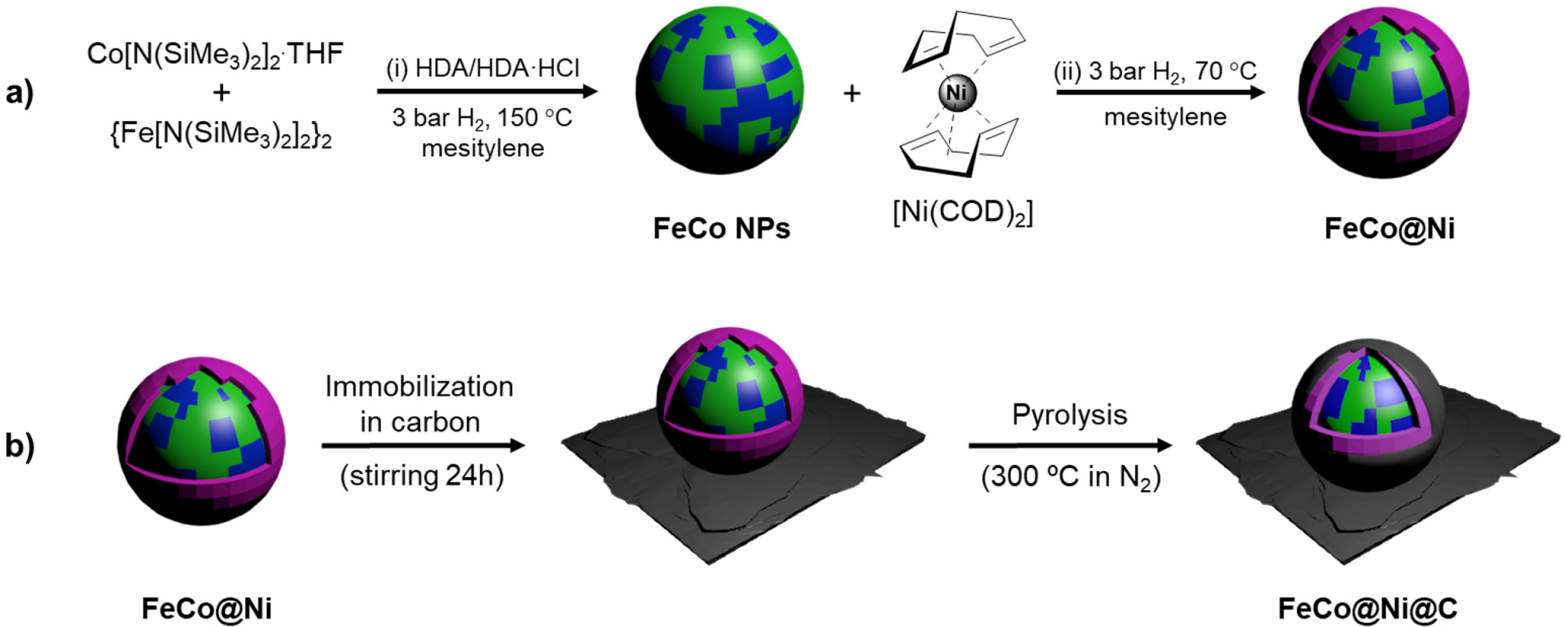
Synthesis of (a) **FeCo@Ni** and (b) **FeCo@Ni@C**.

The synthesis of core–shell **FeCo@Ni** NPs is based on a two-step synthetic method using an organometallic approach. (i) First, **FeCo** NPs stabilized with a mixture of hexadecylamine (HDA) and hexadecylammonium chloride (HDA·HCl) were prepared through the controlled decomposition of {Fe[N(SiMe_3_)_2_]_2_}_2_ and Co[N(SiMe_3_)_2_]_2_·THF under 3 bar H_2_ following a previously reported procedure.^35^ (ii) Once **FeCo** NPs were obtained, the nickel shell was formed by a second decomposition process. More specifically, 1 equiv of Ni(COD)_2_ was decomposed under H_2_ at 70 °C in the presence of **FeCo** NPs dispersed in mesitylene (Figure 1a; for more details, see the section FeCo@Ni), leading to core–shell **FeCo@Ni** NPs that displayed a theoretical metal composition of Fe_25_Co_25_Ni_50_. The experimental Fe, Co, and Ni contents of **FeCo@Ni** were determined by ICP atomic emission spectroscopy (ICP-AES) as 27.9, 21.0, and 35.2 wt %, respectively, which corresponded to a NP composition of Fe_33_Co_25_Ni_42_. Here, the total metal content was 84 wt %, very close to that observed by thermogravimetric analysis (75.5 wt %).

To prevent the sintering of **FeCo@Ni** NPs during the magnetically induced catalysis, the NPs were encapsulated into carbon by adapting a recently reported procedure.^17,36,37^ More specifically, **FeCo@Ni** NPs were covered by a cracked carbon layer (**FeCo@Ni@C**) through a pyrolysis process (300 °C for 2 h at a rate of 10 °C/min) (Figure 1b). The detailed synthetic procedure is described in the Experimental Section (see Synthesis of FeCo@Ni and FeCo@Ni@C NPs). The most significant difference of this modified encapsulation process was the pyrolysis temperature, which was 300 °C instead of 600 °C. This lower pyrolysis temperature was chosen for two reasons: (i) to preserve the core–shell structure of the **FeCo@Ni** NPs, since after pyrolysis at higher temperatures the nickel shell is lost and alloy-type NPs are formed(*vide infra*), and (ii) to contain a larger number of cracks or gaps in the carbon layer, which is important for the substrates to access the active metal sites. The higher number of cracks at 300 °C was confirmed by performing a digestion experiment with H_2_SO_4_ (see Digestion Experiments).^38,39^ Metal contents of **FeCo@Ni@C** were also determined by ICP-AES to be 2.9 wt %, Fe, 2.2 wt % Co, and 5.1 wt % Ni, corresponding to a NP composition of Fe_28_Co_21_Ni_50_; these values were close to the nominal values of 2.5 wt % Fe, 2.5 wt % Co, and 5.0 wt % Ni.

**Figure 2:**
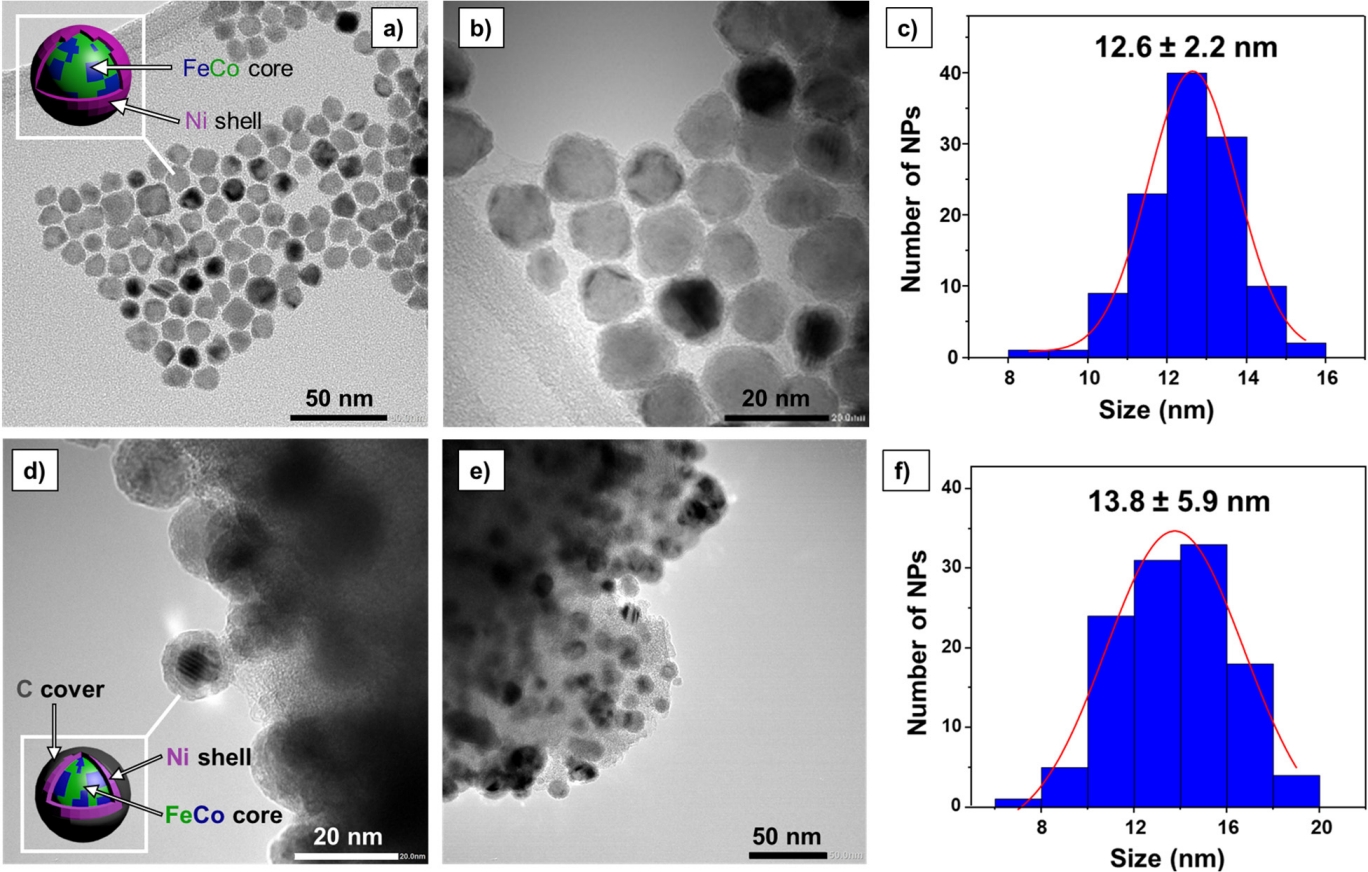
TEM images and size distribution histograms of **FeCo@Ni** (top) and **FeCo@Ni@C** (bottom).

Transmission electron microscopy (TEM) analysis of **FeCo@Ni** NPs showed well-dispersed nanoparticles with a size distribution of 12.6 ± 2.2 nm (Figure 2, a–c). The size and morphology of the **FeCo@Ni@C** NPs were comparable to those found in the MagNPs before encapsulation, presenting a main diameter of 13.8 ± 5.9 nm (Figure 2, d–f). However, after the encapsulation process, the dispersion in the size distribution slightly increased due to the formation of larger nanoparticles during the pyrolysis step.

**Figure 3:**
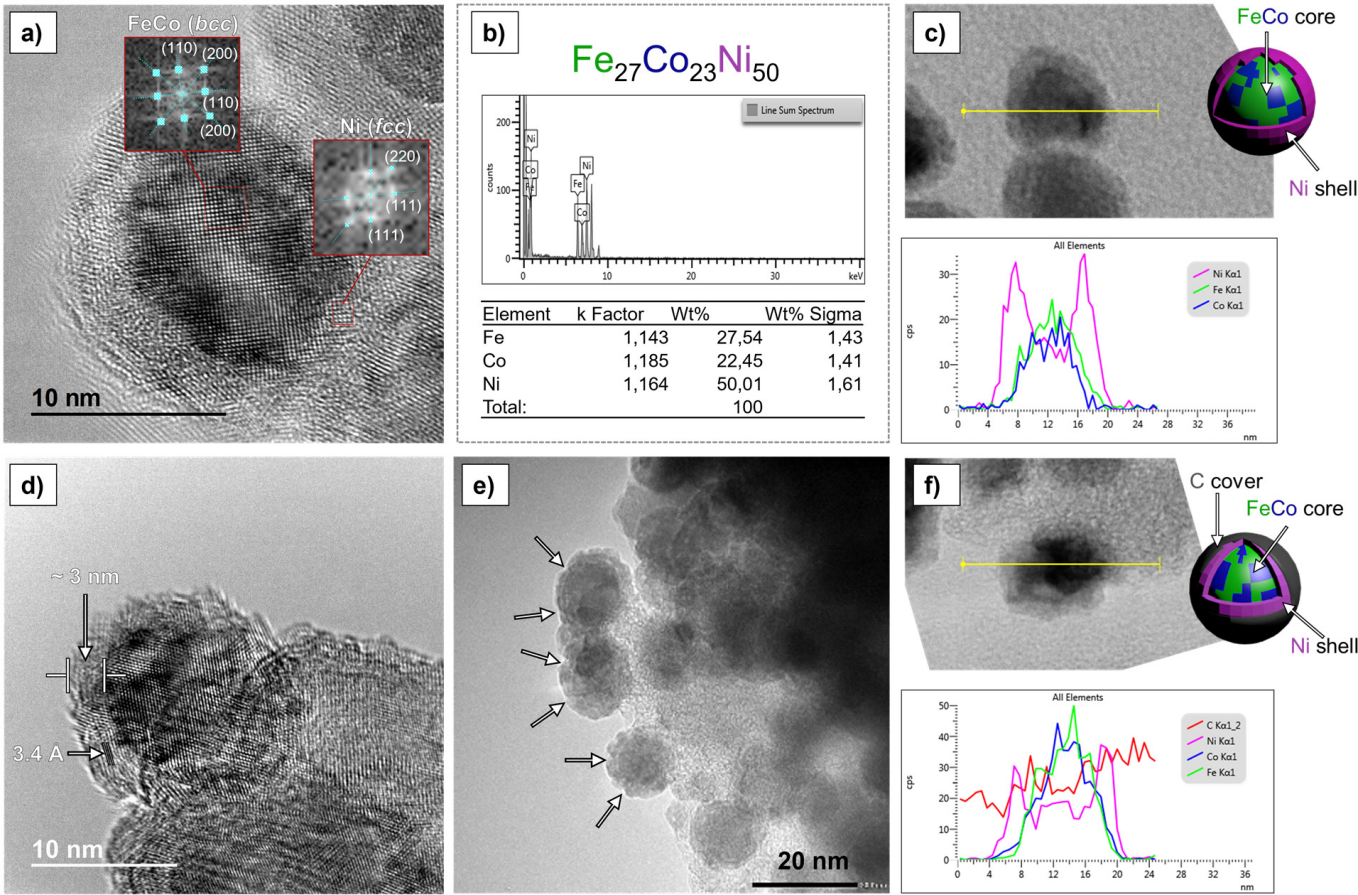
(a) HRTEM image of **FeCo@Ni**. The Fourier analysis of this images confirms that the FeCo core exhibits a *bcc* structure and the Ni shell exhibits an *fcc* structure. (b) Relative composition profile of **FeCo@Ni**. (c) STEM-BF image and EDX line scan profile of **FeCo@Ni**. (d) HRTEM image of **FeCo@Ni@C** highlighting the separation of the carbon layers (3.4 Å) and the thickness of the carbon coat (3 nm). (e) HRTEM image of **FeCo@Ni@C**, with the cracks of the carbon layer highlighted by arrows. (f) STEM-BF image and EDX line scan profile of **FeCo@Ni@C**.

The compositions and crystallinities of **FeCo@Ni** and **FeCo@Ni@C** were investigated by high-resolution TEM (HRTEM) and scanning transmission electron microscopy bright field (STEM-BF) coupled with energy-dispersive X-ray (EDX) spectroscopy. HRTEM micrographs of **FeCo@Ni** indicated the existence of crystalline NPs with an FeCo core and a Ni shell. The FeCo core exhibited a body-centered cubic (*bcc*) structure, and the nickel shell exhibited a face-centered cubic (*fcc*) structure, which is typical of metallic Ni (Figure 3a). Likewise, HRTEM images of **FeCo@Ni@C** showed the same crystalline core–shell MagNPs covered with a thin layer of carbon (∼3 nm thick) (Figure 3d). The distance between the carbon layers was 3.4 Å (Figures 3d), which corresponds to carbon with a turbostratic structure. A large number of cracks could be found in this carbon cover, indicating that substrates have access to the nanoparticle surface (Figures 3e). Despite the presence of these gaps or cracks in **FeCo@Ni@C**, the carbon layer prevents the sintering of the MagNPs at the elevated temperatures reached during the magnetically induced catalytic reactions. The Raman spectrum of **FeCo@Ni@C** NPs (see Figure S1 in SI section S1) shows two bands at 1353 (D band) and 1602 cm^–1^ (G band), which are associated with a disordered carbon material.^40^

The core–shell structures of **FeCo@Ni** and **FeCo@Ni@C** was confirmed by STEM-BF imaging coupled with EDX analysis. In particular, the EDX line scan profile and elemental mapping of **FeCo@Ni** corroborate the core–shell nature of these MagNPs (Figure 3b and c and Figure S2 in SI section S2). The latter shows **FeCo@Ni** NPs with an atomic composition of Fe_27_Co_23_Ni_50_, which matches very well with the nominal composition (Fe_25_Co_25_Ni_50_) and is close to the observed ICP results (*vide supra*). Likewise, EDX analysis of **FeCo@Ni@C** showed FeCo core–Ni shell nanoparticles embedded into a carbon layer (Figure 3f and Figure S3 in SI section S2). Additionally, using STEM-BF coupled to EDX, we also observed the structural transformation of core–shell **FeCo@Ni** NPs into alloy-type NPs at a high pyrolysis temperature. After a pyrolysis process at 600 °C, we could no longer observe the nickel shell in the EDX composition profile of **FeCo@Ni@C** (see Figure S4 in SI section S2), confirming the formation of alloy FeCoNi NPs encapsulated in carbon (**FeCoNi@C**) at a high temperature.

X-ray powder diffraction (XRD) also revealed that the nanoparticles were well-crystallized according to the centered-cubic metal structure. The XRD diffractogram of **FeCo@Ni** NPs exhibits peaks corresponding to *bcc*-FeCo and *fcc*-Ni, with a small amount of Ni oxide (NiO) (see Figure S6 in SI section S3). After the carbon-coating process, the (002) peak of the carbon material appears at 2θ = 31.1° (see Figure S7 in SI section S3). Here, the cubic FeCo and Ni peaks can be observed together with the FeCoO_*x*_ and NiO peaks, but with quite low intensities due to the low metal content (∼10 wt %). Therefore, XRD confirms the partial oxidation of **FeCo@Ni@C** after exposure to air due to the cracked carbon layer.

**Figure 4:**
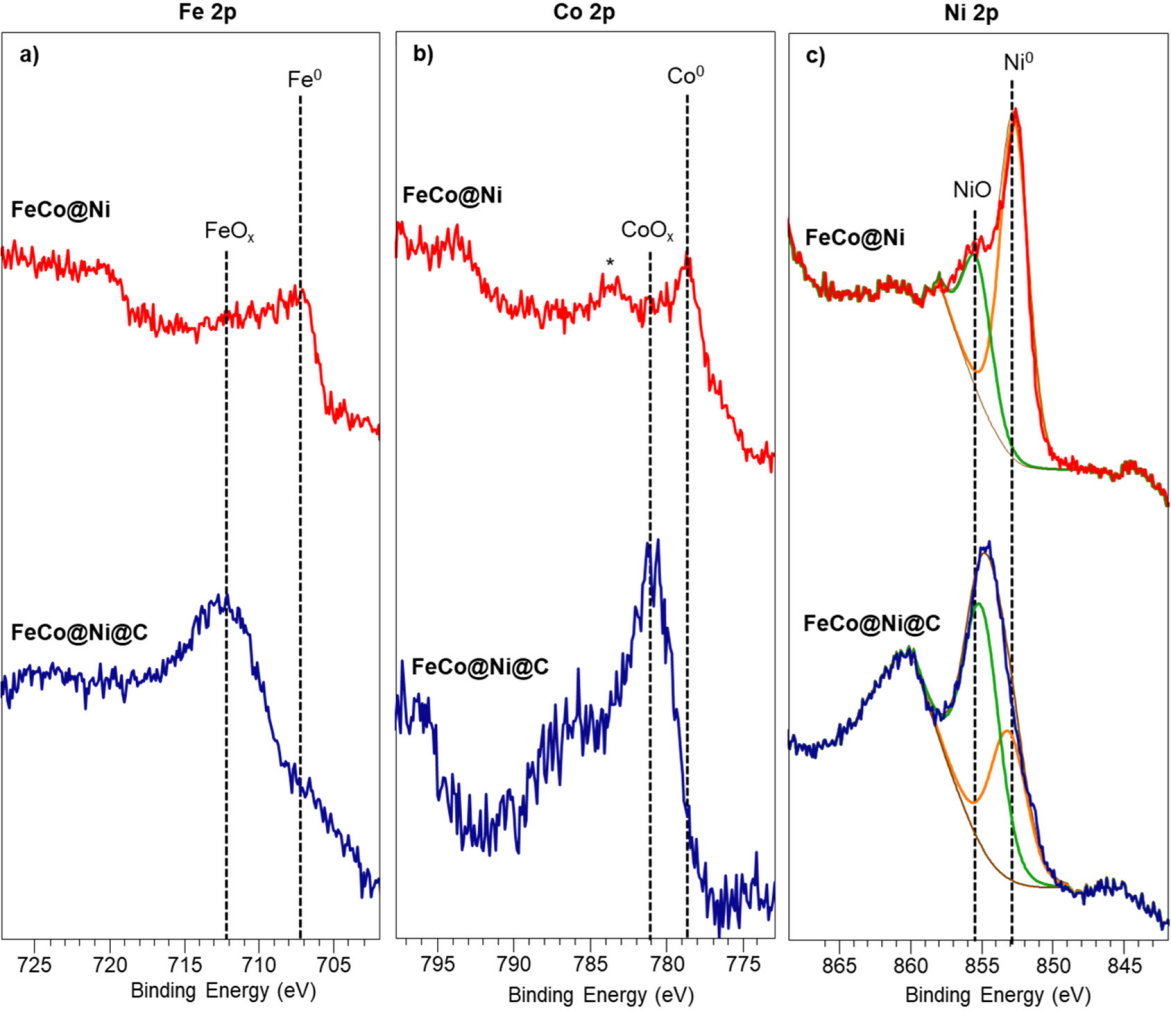
XPS spectra of the (a) Fe 2p, (b) Co 2p, and (c) Ni 2p areas of **FeCo@Ni** (top, red) and **FeCo@Ni@C** (bottom, blue). The asterisk (*) corresponds to the loss feature.

The oxidation states of **FeCo@Ni** NPs before and after the encapsulation were investigated by X-ray photoelectron spectroscopy (XPS). Figure 4a shows the Fe 2p area of **FeCo@Ni** NPs before and after encapsulation. The spectrum of the colloidal **FeCo@Ni** NPs presents a main peak at 707.6 eV that corresponds to Fe^0^. However, after encapsulation and exposure to air, iron was oxidized to FeO_*x*_, since the spectrum exhibited a signal at 712.1 eV characteristic of iron oxide.^41^ A similar pattern was observed in the Co 2p region, where **FeCo@Ni** NPs were mostly reduced (781.1 eV) before encapsulation but were practically oxidized (778.7 eV) after encapsulation in carbon (Figure 4b). Finally, the oxidation state of the nickel shell was studied by analyzing the Ni 2p area. The Ni shell of the as-synthesized **FeCo@Ni** NPs was mostly formed by metallic nickel (852.7–853.2 eV), but after the encapsulation and exposure to air, the percentage of NiO increased (855.1–855.3 eV) (Figure 4c). More specifically, from the analysis of the Ni 2p XPS areas after deconvolution, we can assume that the nickel shell of **FeCo@Ni** is formed by approximately 72% Ni^0^ and 28% NiO, whereas that of **FeCo@Ni@C** is formed by 38% Ni^0^ and 62% NiO.

The magnetic properties of **FeCo@Ni** and **FeCo@Ni@C** NPs were studied by vibrating-sample magnetometry (VSM) using a magnetic field up to 3 T at 300 and 5 K. The saturation magnetization (*M*_s_) and coercive field (μ_0_*H*_c_) values were obtained from the corresponding hysteresis cycles (see Figure S8 in SI section S4). For **FeCo@Ni**, the *M*_s_ was found to be 162 A·m^2^/g at 300 K and 168 A·m^2^/g at 5 K. These *M*_s_ values are lower than those reported for FeCo (230 A·m^2^/g),^42^ probably due to the incorporation of the Ni shell. The *M*_s_ values of **FeCo@Ni@C** were notably lower (65 A·m^2^/g at 300 K and 80 A·m^2^/g at 5 K) due to both the partial oxidation of the MagNPs and their encapsulation into carbon. This encapsulation is known to lead to heating agents with higher stabilities but lower *M*_s_.^17^ The partial oxidation of **FeCo@Ni@C** was corroborated by the existence of a noticeable *exchange bias* field in the hysteresis loops at 5 K, where it was shifted along μ_0_*H*_c_ axis due to the coupling between antiferromagnetic and ferromagnetic layers.^43^

Finally, the heating capacities of **FeCo@Ni** and **FeCo@Ni@C** were estimated by determining the specific absorption rate (SAR) via calorimetry using an already described procedure (see SI section S5).^5,6,44^ The SAR of **FeCo@Ni** NPs was determined in mesitylene due to their good dispersibility in this solvent (see Figure S9a in SI section S5). In this case, the MagNPs started to heat after the application of an alternating magnetic field (μ_*0*_*H*_rms_) of 19 mT with a frequency (*f*) of 100 kHz. The heating power of **FeCo@Ni** measured at 47 mT was 620 W/g. This value is much lower than the reported one of 1600 W/g for FeCo NPs.^6^ This is likely due to the incorporation of the nickel shell, which changes the magnetic anisotropy of the NPs and thus their heating capacity.^45−47^ On the other hand, after the carbon-coating process, **FeCo@Ni@C** started heating at lower amplitudes (14.3 mT) but only reached 82 W/g at 47 mT (see Figure S9b in SI section S5). This agrees with their lower saturation magnetization and coercivity compared to **FeCo@Ni** NPs and is due to the encapsulation of the NPs into carbon and their partial oxidation. The carbon encapsulation makes it difficult to organize the NPs in “chains” following the direction of the magnetic field, which could explain their lower heating power together with their lower *M*_s_.^48,49^ Despite the lower SAR values of the **FeCo@Ni** and **FeCo@Ni@C** NPs, their heating powers are sufficient to heat the systems and carry out magnetic catalysis in solution (*vide infra*).

### Catalytic Studies

**Figure 5:**
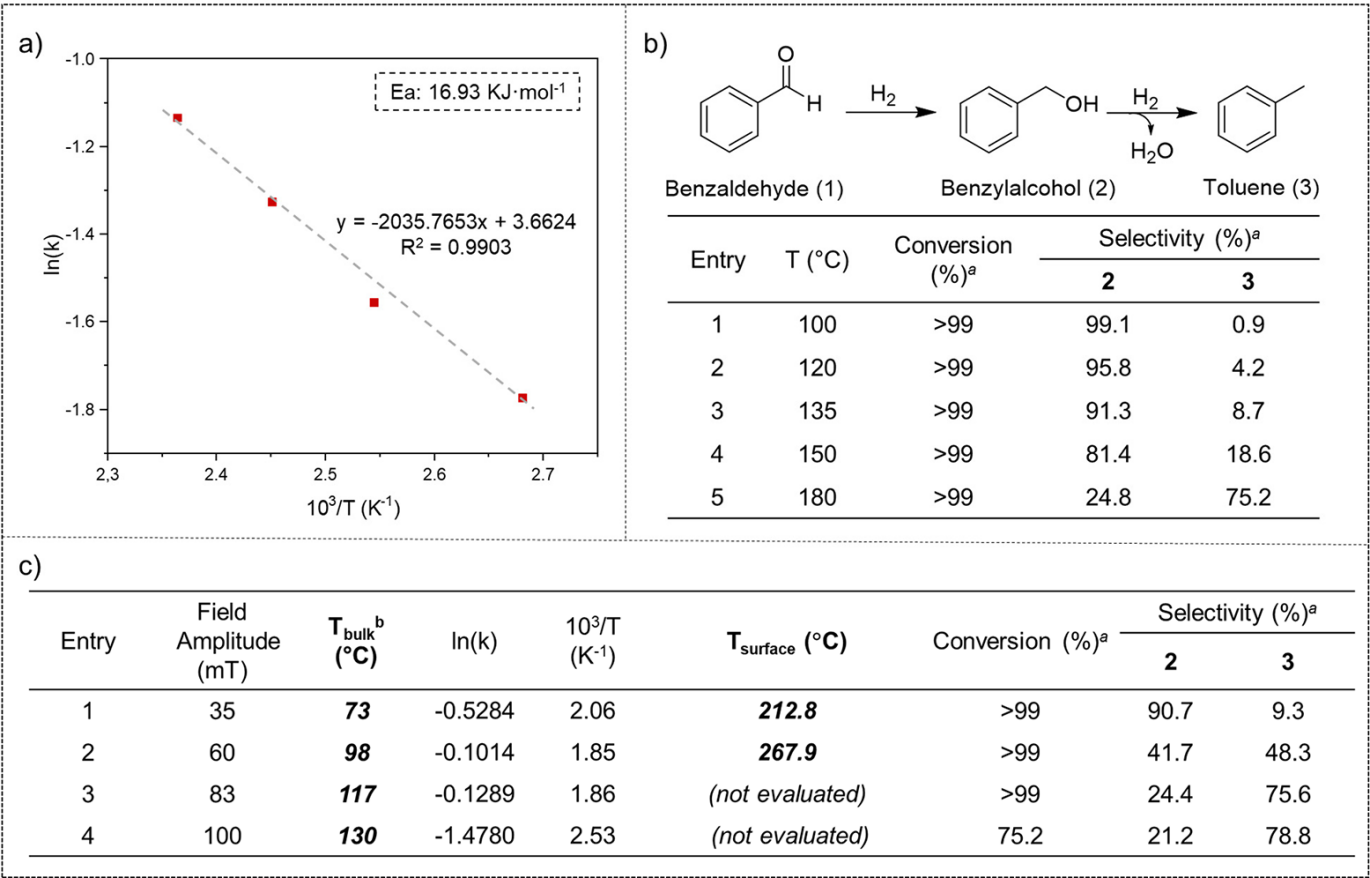
(a) Arrhenius plot of the hydrogenation of benzaldehyde using conventional heating and **FeCo@Ni**. (b) Hydrogenation of benzaldehyde catalyzed by **FeCo@Ni** NPs using conventional heating at different temperatures. (c) Determination of the surface temperature during the hydrogenation of benzaldehyde catalyzed by **FeCo@Ni** NPs using magnetically induced heating at different field amplitudes. ^a^ Reactions conditions are as follows: 0.68 mmol benzaldehyde, 5 mg of **FeCo@Ni** (∼4 mol % Ni), 3 mL of dioxane, and 3 bar H_2_ for 8 h. Conversions and selectivities were determined by GC using dodecane as the internal standard and confirmed by GC-MS. ^b^ The temperature of the bulk was measured by a fiber-optic temperature sensor.

To estimate the surface temperature (*T*_surf_) of **FeCo@Ni** and **FeCo@Ni@C** during the magnetically induced catalysis in solution, we studied the temperature dependence of the catalytic performances of non-coated and carbon-coated MagNPs in the hydrogenation of benzaldehyde in dioxane. We used benzaldehyde as a model substrate since it has two potentially reducible functional groups (*i.e*., carbonyl and an aromatic ring) and it can be hydrodeoxygenated producing the corresponding alkyl compound (*i.e*., toluene). Once the model reaction was chosen, a correlation between the conversion and the temperature was established through the Arrhenius equation using conventional heating. Then, by introducing to this Arrhenius plot the kinetic constant values obtained under magnetic induction catalysis, we were able to determine the value of *T*_surf_ at different magnetic fields. The hydrogenation of benzaldehyde (**1**) under 3 bar H_2_ using conventional heating and **FeCo@Ni** NPs as catalyst enabled us to correlate the temperature and the conversion via the corresponding Arrhenius equation, as demonstrated previously.^16^ Plotting the initial rates (*k*) for the hydrogenation of benzaldehyde (**1**) to benzyl alcohol (**2**) at different temperatures (between 100 and 150 °C; see Figures S10–S13 in SI section S6) produced an Arrhenius plot and the corresponding activation energy (*E*_a_ = 16.93 kJ/mol) (Figure 5a). After 20 h, the conventionally heated reactions were practically selective toward the hydrogenation product benzyl alcohol (**2**) (Table of Figure 5b). At 180 °C, the selectivity changes, and the HDO product toluene (**3**) becomes the main product (see Figure S14 in SI section S6). This point was not included in the Arrhenius plot because the hydrogenation of benzaldehyde was slower than that at 150 °C due to the high reaction temperature and the consequent deactivation of these non-coated **FeCo@Ni** NPs by agglomeration (see Figure S28 in SI section S7). Then, by introducing the initial rate values obtained by magnetically induced catalysis (see Figures S15–S18 in SI section S6) to the Arrhenius plot of Figure 5a, we were able to estimate the *T*_surf_ of **FeCo@Ni** during the magnetic-induced hydrogenation of benzaldehyde (Figure 5c). This estimation worked quite well at low and medium field amplitudes, such as 35 or 60 mT, where the estimated surface temperatures were *ca*. 213 and 268 °C, respectively (table of Figure 5c, entries 1 and 2). These *T*_surf_ values were well above the bulk temperature of the solution (*T*_bulk_) measured by a fiber-optic temperature sensor (see the Experimental Section),^50^ demonstrating the presence of a higher local temperature at the catalyst surface. However, although *T*_bulk_ increased at higher field amplitudes (table of Figure 5c, entries 3 and 4), the conversion values at early reaction times (*k*) were lower than expected; hence, the estimated values of *T*_surf_ were not accurate in these cases. This was due to the agglomeration or coalescence of the **FeCo@Ni** NPs observed at high magnetic fields (see Figure S29 in SI section S7), which reduced their catalytic activity. Similar aggregation and the consequent loss of activity were observed for colloidal Fe_2.2_C@Ru NPs during the magnetically induced hydrodeoxygenation of acetophenone in solution.^16^

The hydrogenation of benzaldehyde (**1**) catalyzed by **FeCo@Ni** NPs using magnetically induced heating at different field amplitudes produced a mixture of benzylalcohol (**2**) and toluene (**3**) (table of Figure 5c). This clearly indicates that both hydrogenation and HDO processes take place under the magnetic field and also points out that *T*_surf_ of **FeCo@Ni** is higher than the measure value of *T*_bulk_. For example, at 60 mT and a bulk temperature of 98 °C, the conversion after 8 h was >99%, with 41.7% selectivity toward benzylalcohol (**2**) and 48.3% selectivity to toluene (**3**) (table of Figure 5c, entry 2). The higher activity and the different selectivity of this reaction compared to those performed at 100 °C under conventional heating (table of Figure 5b, entry 1) confirm the much higher temperature at the nanoparticle surface. Interestingly, at low field amplitude (*i.e*., 35 mT), the main product was benzylalcohol (**2**) (selectivity of 90.7%), but at higher field amplitude (*i.e*., 83 mT), the major product was toluene (**3**) (selectivity of 75.6%) (table of Figure 5c, entries 1 and 3). Taking into account these results, it is possible to control the product distribution by adjusting the amplitude of the alternating magnetic field.

**Figure 6:**
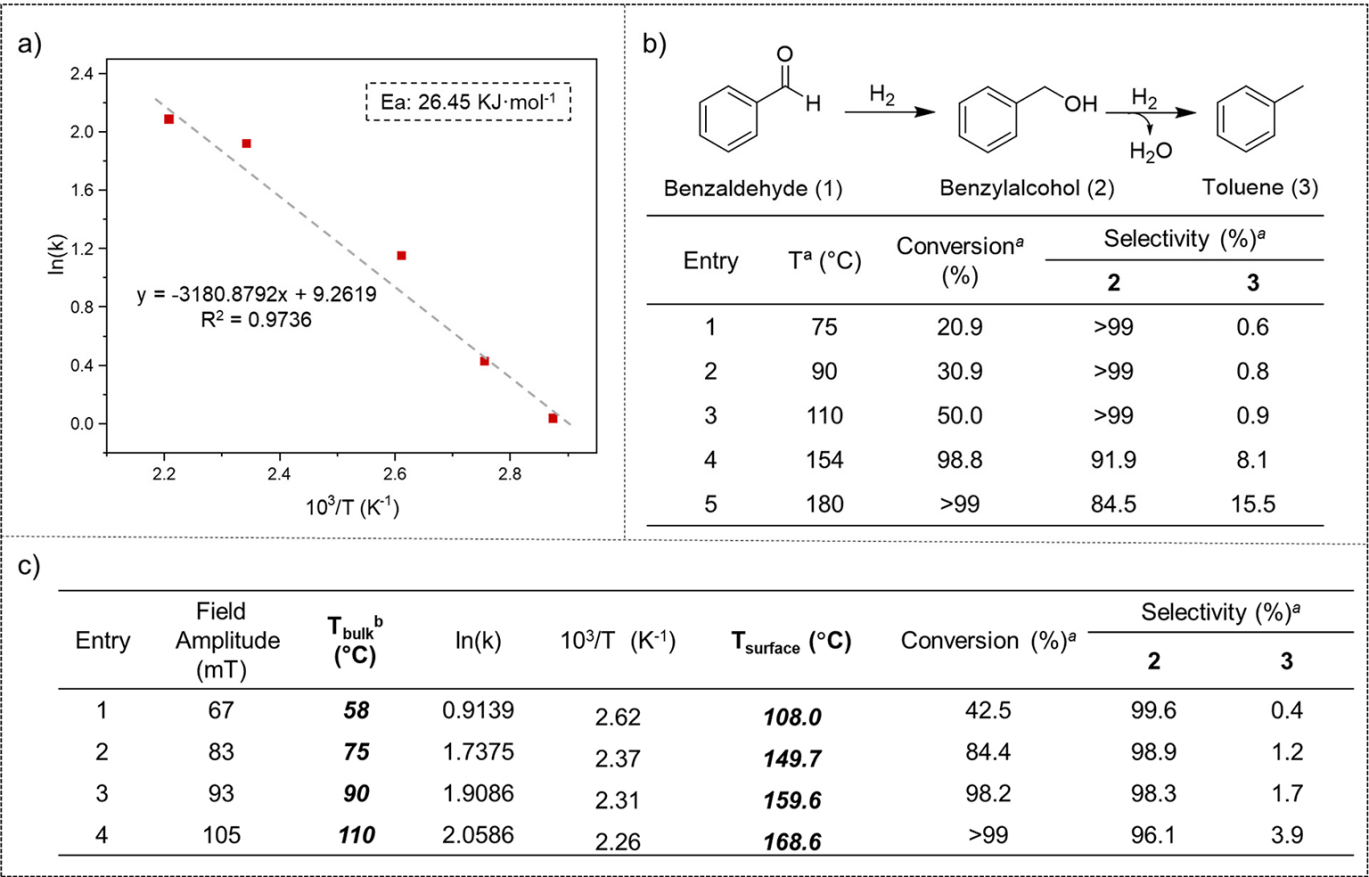
(a) Arrhenius plot of the hydrogenation of benzaldehyde using conventional heating and **FeCo@Ni@C**. (b) Hydrogenation of benzaldehyde catalyzed by **FeCo@Ni@C** using conventional heating at different temperatures. (c) Determination of the surface temperature during the hydrogenation of benzaldehyde catalyzed by **FeCo@Ni@C** using magnetically induced heating at different field amplitudes. ^a^ Reactions conditions are as follows: 0.68 mmol benzaldehyde, 30 mg of **FeCo@Ni** (∼4 mol % Ni), 3 mL of dioxane, and 3 bar H_2_ for 20 h. Conversions and selectivities were determined by GC using dodecane as internal standard and confirmed by GC-MS. ^b^ The temperature of the bulk was measured by a fiber-optic temperature sensor.

Benzaldehyde hydrogenation at different temperatures and magnetic fields was also employed to determine the *T*_surf_ of the carbon-coated **FeCo@Ni@C** NPs under magnetic induction heating. First, we obtained the Arrhenius equation and the corresponding activation energy (*E*_a_ = 26.45 kJ/mol) for the hydrogenation of benzaldehyde toward benzyl alcohol under 3 bar H_2_ using conventional heating between 75 and 180 °C (see Figure 6a and Figures S19–S23 in SI section S6). At long reaction times (20 h), the conventionally heated reactions catalyzed by **FeCo@Ni@C** also produced mostly benzyl alcohol (**2**), but with much lower activities than those catalyzed by **FeCo@Ni** (table of Figure 6b). Indeed, when comparing the activation energies obtained for the hydrogenation of benzaldehyde catalyzed by **FeCo@Ni** and **FeCo@Ni@C** to produce benzyl alcohol, we observed a lower value for the process catalyzed by the non-encapsulated MagNPs (Figures 5a and 6a). The higher catalytic activity observed for the **FeCo@Ni** NPs is understandable, since the number of accessible active surface sites is much higher than that in the case of **FeCo@Ni@C**, where the nanocatalysts are partially encapsulated in carbon.

By introducing the initial rate values of benzyl alcohol (**2**) formation obtained by magnetically induced catalysis (see Figures S24–S27 in SI section S6) to the Arrhenius equation determined by thermal heating (Figure 6a), we calculated the *T*_surf_ of **FeCo@Ni@C** as a function of the applied magnetic field (table of Figure 6c). In this case, the temperature of the NP surface increased linearly over the entire range of magnetic field amplitudes, and a *T*_surf_ of 168.6 °C was reached at 105 mT. Here, we did not observe a decrease of *T*_surf_ at high magnetic fields like in the case of non-coated **FeCo@Ni** NPs, mainly due to the high stability against agglomeration that the encapsulation in carbon confers to the MagNPs. Again, we could experimentally demonstrate that the *T*_surf_ of this magnetic nanocatalyst was well above of the *T*_bulk_ under magnetic excitation, which allowed us to reach high temperatures at the surface of the catalyst (“hot spots”) in a colder environment. Although the magnetically induced hydrogenation of benzaldehyde (**1**) catalyzed by **FeCo@Ni** NPs produces a mixture of benzyl alcohol and toluene (table of Figure 5c), **FeCo@Ni@C** mostly yields the hydrogenation product (Ttble of Figure 5c). At only 105 mT (the maximum field amplitude that we could reach with the equipment), *ca*. 4% toluene was observed. The lower estimated surface temperature compared to that of **FeCo@Ni** explains this selectivity toward the hydrogenation process. At the same time, however, the higher activity of **FeCo@Ni@C** at 93 mT and *T*_bulk_ = 90 °C (table of Figure 6c, entry 3) compared to that in the reaction performed with conventional heating at 90 °C (table of Figure 6b, entry 2) again indicates the higher temperature at the nanoparticle surface compared to that in the bulk solvent.

**Figure 7:**
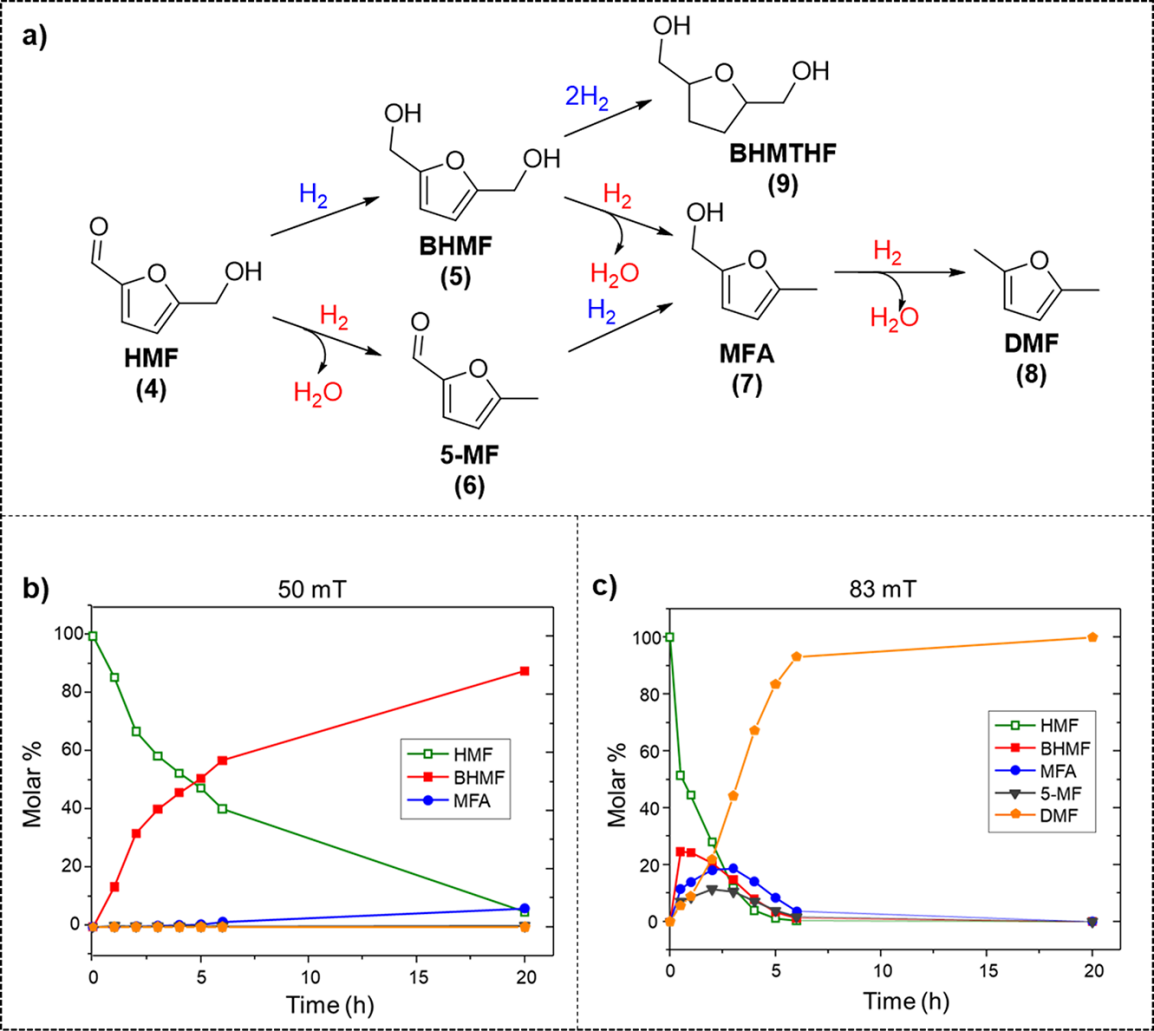
(a) Catalytic transformation of hydroxymethylfurfural (HMF) using **FeCo@Ni** NPs at (b) 50 and (c) 83 mT.

On the basis of the heating capacity of these materials in solution, we decided to evaluate their catalytic performances with biomass-derived oxygenated substrates, such as 5-hydroxymethylfurfural (HMF). HMF, which can be directly obtained from the thermal or chemical treatment of biomass, is a key biomass-derived platform chemical^51^ and an important building block for a broad range of applications in the areas of polymers, fine chemicals, and fuels. For example, HMF (**4**) can be hydrogenated to 2,5-bishydroxymethylfuran (BHMF, **5**) and 2,5-bishydroxymethyltetrahydrofuran (BHMTHF, **9**), which are attractive monomers for the production of polymers. In addition, HMF can be also converted by hydrogenolysis to 2,5-dimethylfuran (DMF, **8**), which is a promising biofuel. Figure 7 shows the kinetic behavior for HMF conversion using **FeCo@Ni** NPs at field amplitudes of 50 and 83 mT under 3 bar H_2_ in 1,4-dioxane. At 50 mT, which correspond to a *T*_bulk_ of 75 °C, the conversion of HMF essentially proceeds parallel to the formation of the diol BHMF (**5**). Only small amounts of the HDO products were observed after 20 h of reaction (Figure 7b). However, at 83 mT (*T*_bulk_ = 106 °C), the selectivity of the reaction was totally different; after 3 h, the major product was DMF (**8**), and the reaction was found to be fully selective to the same product at completion (Figure 7c). More specifically, after 20 h at a magnetic field of 50 mT, the conversion reached 95%, with 92.8% selectivity toward BHMF (**5**), and only small amounts of HDO products 5-methylfurfural (**6**) and 2-hydroxymethyl-5-methylfuran (**7**) were produced (Table 1, entry 1). On the contrary, at a higher field amplitude (*i.e*., 83 mT), total conversion to DMF (**8**) was observed (Table 1, entry 2). **FeCo@Ni** NPs showed a complete selectivity change between the hydrogenated product, BHMF (**5**), and the hydrodeoxygenated product, DMF (**8**), with the increase of the magnetic field applied. Thus, by adjusting the field amplitude during the magnetically induced catalysis, it was possible to control the product distribution.

**Table 1:** Hydrogenation of Hydroxymethylfurfural (HMF) Catalyzed by **FeCo@Ni** and **FeCo@Ni@C** Using Magnetically Induced Heating

						selectivity^b^			
entry	catalyst	field (mT)	solvent	*T*_bulk_^a^ (°C)	conversion^b^ (%)	BHMF	5-MF	MFA	DMF
1	FeCo@Ni	50	dioxane	75	95.0	**92.8**	0.5	6.7	0
2	FeCo@Ni	83	dioxane	106	>99				**100**
3	FeCo@Ni	83	water	99	24.0	**100**			
4	FeCo@Ni@C	60	dioxane	81	19.0	**95.0**	1.4	3.6	
5	FeCo@Ni@C	100	dioxane	143	>99	4.8	2.5	10.9	**81.8**
6	FeCo@Ni@C	100	water	95	93.4	**100**			
7	FeCo@Ni@C	100	water/dioxane 1:2	115	90.9	**96.9**	0.6	2.2	0.3
8	FeCo@Ni@C	100	water/dioxane 1:8	130	95.5	**83.8**	1.3	8.7	6.2
9	FeCo@Ni@C	100	water/dioxane 1:32	136	>99	**79.8**	0.4	10.3	9.5

a The temperature of the bulk was measured by a fiber-optic temperature sensor.

b Conversions and selectivities at 20 h. Reactions conditions are as follows: 0.5 mmol HMF, 5 mg of **FeCo@Ni** (∼6 mol % Ni), 50 mg of **FeCo@Ni@C** (∼8 mol %), 3 mL of solvent, and 3 bar H_2_. Conversions and selectivities were determined by GC using dodecane as the internal standard and confirmed by GC-MS.

Going one step further, we decided to carry out the magnetically induced reduction of HMF in aqueous media, a very challenging reaction due to the poor solubility and low stability of ligand-stabilized MagNPs in water. Actually, **FeCo@Ni** exhibited a completely different activity and selectivity in water. After 20 h at 83 mT (*T*_bulk_ = 99 °C), in water the conversion was only 24% with 100% selectivity to BHMF (**5**), whereas in dioxane there was a complete conversion to DMF (**8**) (Table 1, entries 2 and 3, respectively). This lack of activity in water is due to several factors, including oxidation and the high degree of aggregation of non-supported **FeCo@Ni** NPs undergo in water (see Figure S32 in SI section S8), where the nanoparticles are still aggregated even after being sonicated during 1 h. On the contrary, **FeCo@Ni** NPs are uniformly dispersed in dioxane after 10 min of sonication (see Figure S33 in SI section S8).

The more stable and robust **FeCo@Ni@C** catalyst was also evaluated in the hydrogenation of HMF in both aqueous and organic media. After 20 h in dioxane at 60 mT (*T*_bulk_ = 81 °C), the conversion was poor, but HMF was selectively hydrogenated to BHMF (**5**) (95% selectivity at 19% conversion) (Table 1, entry 4). However, at larger field amplitude (*i.e*., 100 mT and *T*_bulk_ = 143 °C) the conversion was complete and the selectivity of the reaction again switched toward DMF (**8**) (Table 1, entry 5). Here, in a manner similar to that of non-encapsulated **FeCo@Ni** NPs in dioxane, the activity and product distribution are controlled by the magnetic field applied during the catalysis. A different scenario was observed when water was used as solvent in the magnetic reduction of HMF. After 20 h under a magnetic excitation of 100 mT, the only product observed was the diol obtained from the hydrogenation process (Table 1, entry 6). Indeed, HMF was selectively hydrogenated to BHMF (**5**) with a high conversion (93.4%). The aqueous solution exhibited a *T*_bulk_ of 95 °C at 100 mT, which was far from that achieved in dioxane at the same field amplitude (*i.e*., *T*_bulk_ = 143 °C at 100 mT). This difference in the bulk temperature could reflect a difference in the surface temperature and partially explain the different reactivity of **FeCo@Ni@C** depending on the solvent used during the magnetic catalysis (Table 1, entries 5 and 6). The lower *T*_bulk_ achieved in aqueous media is due to the higher specific heat capacity of water (*c*_*p*_ = 4.184 J/kg·K) compared to that of dioxane (*c*_*p*_ = 1.702 J/kg·K). In any case, the high conversion and excellent selectivity obtained in the aqueous transformation of HMF using **FeCo@Ni@C** as catalyst (Table 1, entry 6) are great achievements in magnetically induced catalysis, being the first example reported to date. It is worth mentioning that the catalytic activities of the **FeCo@Ni** and **FeCo@Ni@C** systems prepared herein are comparable to those of other catalytic systems previously reported in the literature (see Table S1 in SI section S9), but with the added advantages of magnetic induction catalysis in water.

To better understand the different selectivities observed depending on the solvent used, the magnetic hydrogenation of HMF was also performed in dioxane/water mixtures. When the reaction was carried out in 2:1 and 8:1 dioxane/water mixtures, the activities and selectivities of **FeCo@Ni@C** were comparable to those observed in neat water, with the main product being BHMF (**5**) (Table 1, entries 7 and 8, respectively). Here, *T*_bulk_ was 115–130 °C, higher than that in aqueous media (*T*_bulk_ = 95 °C). Thus, the change in selectivity is not just a temperature issue. To confirm that, the magnetic hydrogenation of HMF was also conducted in a 32:1 dioxane/water mixture. After only 0.1 mL of water was added to the reaction mixture, a high selectivity toward BHMF (**5**) was observed at *T*_bulk_ = 136 °C (Table 1, entry 9). It was thus found that even a small amount of water in dioxane is enough to change the selectivity of the reaction. The latter results evidence that the high selectivity toward BHMF observed in water is not due to the lower *T*_bulk_. Instead, this selectivity is probably due to an active-site blockage, a consequence of the adsorption of water molecules on the Ni surface or the formation of nickel hydroxide at the surface of the NPs.

Motivated by the promising activity of **FeCo@Ni@C** in aqueous solution, we decided to further investigate its catalytic reactivity in the magnetic reduction of a series of biomass-derived oxygenated compounds such as furfural, levoglucosenone, levulinic acid, and vanillin (Table 2). For example, the hydrogenation of furfural led to a conversion of 81.9% and selectively produced the hydrogenation product (**10**) (Table 2, entry 1). A similar result was found in the hydrogenation of levoglucosenone (**12**) (one of the major products obtained from the pyrolysis of cellulose), where **FeCo@Ni@C** produced a large amount of the totally hydrogenated product (**14**) (Table 2, entry 2). On the other hand, the magnetic conversion of another platform molecule obtained from cellulosic biomass, levulinic acid (**15**), was very low. The hydrogenation and subsequent dehydration of levunilic acid selectively yielded γ-valerolactone (**16**) with a poor conversion (*i.e*., 28.6%) (Table 2, entry 3).^52−54^ Finally, after 20 h at 100 mT, vanillin (**17**) was effectively hydrodeoxygenated into 2-methoxy-4-methylphenol (**19**) (selectivity of 100% at >99% conversion) (Table 2, entry 4). Remarkably, when the applied magnetic field was reduced to 50 mT, we achieved a selectivity of >99% and a conversion of 88.4% in the hydrogenation of vanillin into vanillyl alcohol (**18**) (Table 2, entry 6). When the field amplitude is in-between these values (*i.e*., 67 mT), the products contain both vanillyl alcohol (**18**) and 2-methoxy-4-methylphenol (**19**) (Table 2, entry 5). Therefore, exceptional selectivities for both hydrogenation and HDO can be also achieved in water using the same catalyst by changing only the magnetic field applied.

**Table 2:**
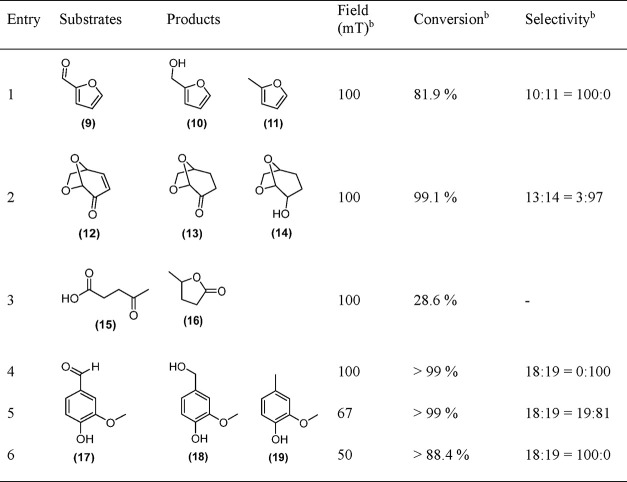
Application of **FeCo@Ni@C** in the Magnetic Hydrogenation of Biomass-Derived Oxygenated Compounds in Water^a^

a Conversion and selectivities at 20 h. Reactions conditions are as follows: 0.5 mmol substrate, 50 mg of **FeCo@Ni@C** (∼8 mol %), 3 mL of water, and 3 bar H_2_.

b Conversions and selectivities were determined by GC using dodecane as standard and confirmed by GC-MS.

**Figure 8:**
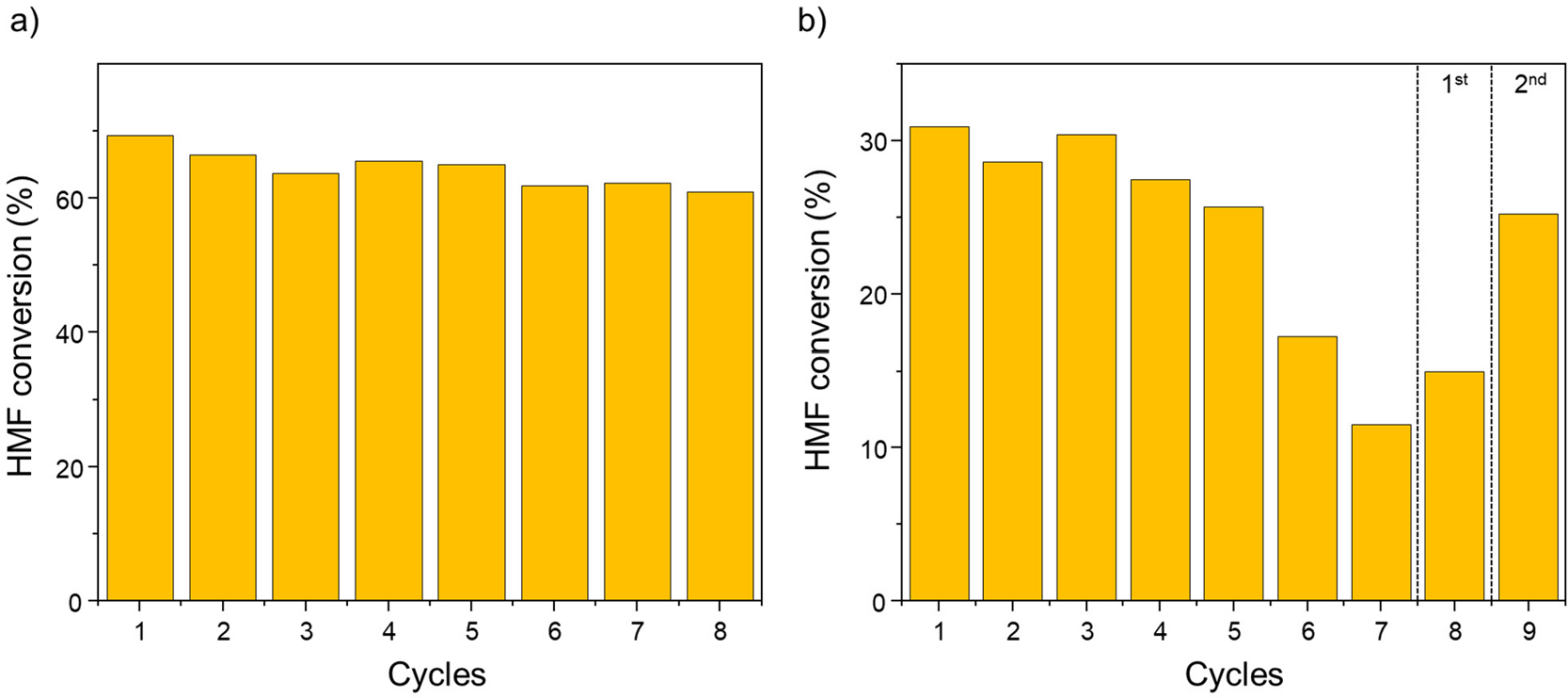
Recycling experiments using **FeCo@Ni@C** in (a) dioxane and (b) water (regeneration treatments after the seventh run are separated by dashed lines). Reaction conditions are as follows: 0.5 mmol HMF, 50 mg of **FeCo@Ni@C** (∼8 mol %), 3 mL of solvent, 3 bar H_2_, and 100 mT.

Finally, to investigate the stability of the carbon-encapsulated core–shell magnetic nanoparticles in dioxane and water, a series of recycling experiments were performed. First, the magnetic hydrogenation of HMF was performed in dioxane multiple times using the same batch of **FeCo@Ni@C**. More specifically, after 4 h of reaction at 100 mT, the catalyst was effortlessly recovered from the reaction media with the help of an external magnet and then washed and dried before the next cycle. Eight consecutive recycling reactions proceeded without any remarkable loss of activity and selectivity (conversions between 69% and 60%) (Figure 8a and Figure S34 in SI section S10). TEM and HRTEM BF-STEM micrographs recorded after the recyclability experiment revealed carbon-encapsulated **FeCo@Ni@C** NPs with a size distribution and a metal composition similar to those of the as-synthesized NPs (see Figure S5 and S30 in SI sections S2 and S7, respectively). The latter demonstrates the recyclability and robustness of **FeCo@Ni@C** under catalytic conditions (100 mT and 3 bar H_2_). Therefore, the encapsulation in carbon remarkably increases the stability of these magnetic nanoparticles, preventing their agglomeration and allowing them to keep both their activity and heating capacity (see Scheme S1 in SI section S11). This encapsulation notably improved the stability of magnetic catalysts in solution, making it possible to reuse them up to eight times.^13^ Later, a similar recyclability study was performed in aqueous media. Seven consecutive reactions were performed using the same **FeCo@Ni@C** batch in water. In this case, the reactivity of **FeCo@Ni@C** NPs remained practically constant during the first four recycles (conversions between 31 and 27%) (Figure 8b and Figure S35 in SI section S10). However, after the fourth run, the conversion progressively decreased until 11%, showing an important loss of activity. Since the TEM analysis revealed the presence of carbon-encapsulated NPs with similar sizes (see Figure S31 in SI section S7), this loss of activity may be due to a gradual blockage caused by the adsorption of water molecules onto the catalyst or a higher degree of oxidation of **FeCo@Ni@C** in water. To check these hypotheses, two different regeneration treatments were performed as follows: (i) After the seventh run, the catalyst was heated at 110 °C under a N_2_ flow over 12 h (to facilitate the desorption of adsorbed molecules) and partially recovered its activity. This indicates that water molecules may act as reaction inhibitors by adsorbing onto the **FeCo@Ni@C** catalyst, as was previously suggested during the catalysis in dioxane/water mixtures. (ii) After being heated at 180 °C under 3 bar H_2_ over 12 h, the catalyst almost recovered its initial activity, suggesting that the Ni surface was also partially oxidized during the magnetic catalysis in water.

## Conclusions

We have efficiently reduced a series of biomass-derived oxygenated compounds by magnetically induced catalysis in both organic and aqueous media using non-coated and carbon-coated core–shell **FeCo@Ni** magnetic nanoparticles as catalysts (**FeCo@Ni** and **FeCo@Ni@C**). A combined characterization study comprising HRTEM, EDX, XRD, XPS, and VSM concluded that **FeCo@Ni** and **FeCo@Ni@C** present FeCo cores and Ni shells with metal compositions close to the nominal value (Fe_25_Co_25_Ni_50_). However, after encapsulation in carbon, the core–shell MagNPs were partially oxidized. We also found that the encapsulation process must occur at a maximum pyrolysis temperature of 300 °C to preserve the core–shell structure and to have a great number of cracks in the carbon layer, which is important for the accessibility of the active metal surface. The surface temperatures (*T*_surf_) of **FeCo@Ni** and **FeCo@Ni@C** during the magnetically induced catalysis were estimated by establishing a correlation between the conversion and the temperature using conventional heating and a model reaction (benzaldehyde hydrogenation). This kinetic approach estimated that the *T*_surf_ values of the magnetic catalysts are much higher than the bulk temperatures of the solutions (*T*_bulk_), which were measured by a fiber-optic temperature sensor. According to the catalytic results, both the activity and the selectivity of **FeCo@Ni** and **FeCo@Ni@C** were highly dependent on the magnetic field applied and the solvent used. For example, in the magnetically induced hydrogenation of HMF over **FeCo@Ni** NPs in dioxane, the main product was BHMF at a low field amplitude (*i.e.*, 50 mT), while the major product was DMF at a higher field amplitude (*i.e.*, 83 mT). Thus, it was possible to control the product distribution by controlling the amplitude of the alternating magnetic field, and we observed for the first time the magnetic-field-controlled selectivity of hydrogenation and hydrodeoxygenation in magnetically induced catalysis. On the other hand, **FeCo@Ni** showed a completely different activity and selectivity in water, mostly due to its poor stability in water. However, the encapsulation in carbon circumvented this limitation and led to stable catalysts suitable for magnetically induced catalysis in both dioxane and water. Indeed, **FeCo@Ni@C** efficiently hydrogenated a series of biomass-derived oxygenated compounds in water, showing its excellent stability. Aside form being a precedent, the aqueous transformation of biomass-derived oxygenated compounds is a great success in magnetically induced catalysis, since it is a very energetically efficient method for performing catalysis in a more sustainable way and transforming real biomass mixtures.

## Experimental Section

### General Considerations and Starting Materials

All the chemical operations were carried out using standard Schlenk tubes or Fischer–Porter bottle techniques or in a glovebox under a N_2_ atmosphere. Mesitylene (99%, extra pure) and 1,4-dioxane (anhydrous, 99.8%) were obtained from Acros Organics and Merk, respectively, then dried on 5 Å molecular sieves and degassed by bubbling Ar for 20 min. The commercial products, hexadecylamine (HDA, 99%) and Ni(COD)_2_ (COD = cyclooctadiene), were obtained from Merk. [Fe{N(SiMe_3_)_2_}_2_]_2_ and [Co{N(SiMe_3_)_2_}_2_(THF)] precursors were obtained from Nanomeps (Toulouse, France). All the catalytic substrates used, such as benzaldehyde (97%), 5-hydroxymethylfurfural (99%), furfural (99%), levunilic acid (98%), vanillin (99%), and dodecane (99%), were obtained from Merk. All the commercial compounds were used as received except for HMF and furfural, which were purified via filtration with an equimolar mixture of silica and alumina and stored in a refrigerator.

#### Transmission Electron Microscopy (TEM) and High-Resolution TEM (HRTEM)

TEM and HRTEM micrographs of **FeCo@Ni** and **FeCo@Ni@C** NPs were obtained after a drop of the corresponding material was suspended on a copper grid. TEM micrographs were acquired at the “Servicio de Microscopía Electrónica” of Universitat Politècnica de València (UPV) using a JEOL JEM 1400Flash instrument operated at 120 kV, with a point resolution of 3.8 Å. HRTEM images were acquired using a JEOL 2100F microscope operated at 200 kV in both the transmission (TEM) and scanning transmission (STEM) modes. EDX and STEM images were obtained using a bright-field (BF) detector. Particles were measurement with ImageJ software, and interplanar spacing and fast Fourier transform (FFT) treatments were performed with Digital Micrograph, ver. 3.7.4.

#### Thermogravimetric Analysis (TGA)

TGA analysis was carried out on a Netzsch TGA/STA 449 F2 Jupiter instrument, with a steam air flow of 100 mL/min and a heating rate of 10 °C/min to reach the maximum temperature of 600 °C.

#### Inductively Coupled Plasma (ICP)

ICP analyses of **FeCo@Ni** and **FeCo@Ni@C** were performed with a Varian 715-ES ICP optical emission spectrometer. Digestion of the carbon-coated catalyst was performed following a recently reported method.^55^ More specifically, 30 mg of the catalyst was diluted with 21 mL of HCl/HNO_3_ (6:1), and the mixture was sonicated for 90 min. Then, the samples were digested at 180 °C for 15 h. Finally, the solution was cooled to room temperature (r.t.) and diluted with water to a final volume of 100 mL.

#### Raman Spectroscopy

Raman spectra were recorded at an excitation wavelength of 514 nm using a Renishaw inVia Raman spectrometer equipped with a CCD detector. The samples (powder) were deposed on an Al support, and a total of 20 acquisitions were taken at a resolution of <4 cm^–1^ for each spectrum in the region between 0 and 3000 cm^–1^.

#### X-ray Photoelectron Spectroscopy (XPS)

XPS spectra of **FeCo@Ni** and **FeCo@Ni@C** were recorded with a SPECS spectrometer equipped with a Phoibos 150MCD-9 multichannel analyzer using non-monochromatic Mg Kα radiation (*h*ν = 1235.6 eV) and Al Kα radiation (*h*ν = 1483.6 eV) from a dual source. The XPS analysis were performed using an analyzer pass energy of 30 eV and an X-ray power of 100 W, and the pressure was maintained under 10^–9^ mbar during the measurements. CASA software was used for the quantification and titration of the spectra, and the binding energy (BE) values were referenced to the C 1s peak (284.8 eV).

#### X-ray Powder Diffraction (XRD)

XRD measurements were performed on a PANalytical Empyrean diffractometer using Co Kα radiation (λ = 0.1789 nm) at 45 kV and 40 mA.

#### Gas Chromatography (GC)

The spectra of benzaldehyde and its hydrogenated products were recorded with an Agilent Technologies 7890A GC system with a flame ionization detector and an HP-5 column (30 m × 250 μm). The instrument was set to an injection volume of 1 μL, an inlet split ratio of 50:1, and a detector temperature of 280 °C. The method used began with an injection temperature of 80 °C for 2 min. Then, the column is heated at a rate of 10 °C/min until it reaches 160 °C. Finally, the column is heated to 280 °C (30 °C/min) and held there for 1 min. On the other hand, all the catalysis reactions carried out in water (HMF, furfural, levunilic acid, levuglucosenone, and vanillin) were recorded with a Varian CP-3800 system with a Varian CP-8400 automatic injector and a suprawax column. The method used began with an injection temperature of 50 °C. After the temperature was held for 1 min, the column was heated to 240 °C (20 °C/min), and this temperature was maintained for 3.5 min. For the GC measurement of water-based samples, a 200 μL aliquot of the sample was taken and diluted with 800 μL of isopropanol with 0.1 mmol dodecane, which was used as the standard.

#### Gas Chromatography–Mass Spectrometry (GC-MS)

GC-MS spectra were acquired in an Agilent 6890N chromatograph coupled to an Agilent 5973N mass selective detector equipped with a HP5-M5 capillary column (30 m × 250 μm). Helium was used as a carrier gas with a constant flow rate of 1.2 mL/min. The temperature program used for all the analyses began at 50 °C for 2 min. Then, the column was heated to 280 °C with a heating rate of 30 °C/min and held there for 15 min.

#### Vibrating-Sample Magnetometer (VSM)

VSM measurementes were carried out using a Quantum Device PPMS Evercool II system. VSM studies of the **FeCo@Ni** NPs were performed on compact powder samples that were prepared and sealed under an argon atmosphere, while those of the **FeCo@Ni@C** catalyst were performed on samples prepared in air.

### Synthesis of **FeCo@Ni** and **FeCo@Ni@C** NPs

#### **FeCo@Ni**

This synthesis followed a two-step procedure. (i) First, bimetallic FeCo NPs were prepared according to a previously reported procedure.^35^ In particular, [Fe{N(SiMe_3_)_2_}_2_]_2_ (150 mg, 0.2 mmol) and [Co{N(SiMe_3_)_2_}_2_(THF) (180 mg, 0.4 mmol)] were reduced under 3 bar H_2_ in the presence of hexadecylamine (HDA; 386 mg, 1.6 mmol) and hexadecylamine hydrochloride (HDA·HCl; 333, 1.2 mmol) at a temperature 150 °C for 24 h in mesitylene (20 mL). After the reaction, a black solution was obtained, wherein the magnetic FeCo NPs were stabilized by HDA and HDA·HCl. (ii) In a second step, Ni(COD)_2_ (220 mg, 0.8 mmol) was added to the solution of FeCo NPs and then reduced under 3 bar H_2_ at 70 °C for 20 h. After the formation of the Ni shell, the **FeCo@Ni** NPs were purified by washing them three times with 5 mL of toluene. The resulting magnetic solid was stored in a glovebox. The sizes of the NPs were measured by TEM for at least 100 nanoparticles, which afforded a mean value of 12.6 ± 2.2 nm (Figure 2c). ICP: Fe, 27.9 wt %; Co, 21.0 wt %; Ni, 35.2 wt %.

#### **FeCo@Ni@C**

In a Schlenk tube, 920 mg of C Norit D10 was dispersed in 15 mL of mesitylene. Then, the carbon dispersion was added to a Fischer–Porter bottle previously charged with a suspension of **FeCo@Ni** NPs (92 mg) in 20 mL of mesitylene. After 24 h of vigorous stirring, **FeCo@Ni** NPs were adsorbed on the activated carbon. After that, carbon-supported **FeCo@Ni** NPs were washed with hexane three times and dried at 60 °C overnight. The resulting black ground powder was transferred into a quartz reactor and placed in a vertical oven for pyrolysis (2 h at 300 °C under N_2_ pressure with a heating ramp if 10 °C/min), producing the **FeCo@Ni** NPs encapsulated in carbon (**FeCo@Ni@C**). The sizes of the carbon-coated NPs was measured by TEM for at least 100 nanoparticles, which afforded a mean value of 13.8 ± 5.9 nm (Figure 2f). ICP: Fe, 2.9 wt %; Co, 2.2 wt %; Ni, 5.1 wt %.

### Digestion Experiments

Following the procedure of Bao and co-workers,^38^ who concluded that CoNi NPs were completely encapsulated in graphene because they were not soluble in a strong acid, we demonstrated that the carbon layer of **FeCo@Ni@C** presented a great number of cracks. Specifically, approximately 10 mg of **FeCo@Ni@C** NPs were practically dissolved in a H_2_SO_4_ solution (2 M, 5 mL) at 80 °C after 2 h. The resulting solutions were analyzed using ICP-AES, and we observed metal contents of 2.6 wt % Fe, 2.1 wt % Co, and 5.1 wt % Ni, which are very close to the theoretical ones (2.5 wt % Fe, 2.5 wt % Co, and 5 wt % Ni). This indicates that practically all metals present in **FeCo@Ni@C** were dissolved due to the presence of a large number of gaps in the carbon layer. On the other hand, when the same digestion experiment was performed on **FeCo@Ni@C** pyrolized at 600 °C, the metal contents were 1.5 wt % Fe, 1.5 wt % Co, and 2.9 wt % Ni, which indicated a lower number of cracks or gaps in the carbon layer.

### Catalytic Reactions

Catalytic experiments were performed in an 80 mL Fischer–Porter reactor with a 160 mm diameter. The reactor was placed at the center of an inductor delivering an AC magnetic field that oscillated at a frequency of 300 kHz, with a root-mean-square (rms) amplitude that could be adjusted between 35 and 105 mT (see SI sections S12 and S13). The induction coil has six turns that are 25 mm wide and 42 mm high. The reactor was filled with the magnetic catalyst (5 mg of **FeCo@Ni** or 50 mg of **FeCo@Ni@C**). For the **FeCo@Ni** catalyst, the Fischer–Porter reactor was filled inside the glovebox to avoid the oxidation of the core–shell nanoparticles. Then, the corresponding substrate (0.68 mmol concentration for benzaldehyde or 0.5 mmol concentration for all the other catalytic substrates) and 3 mL of solvent (dioxane, water, or dioxane/water mixtures) was added, and the atmosphere of the Fischer–Porter was exchanged by carefully pressurizing and depressurizing the reactor with hydrogen three times. After that, the indicated pressure was adjusted (3 bar), and the reaction mixture was introduced in the middle of the coil at the required amplitude and time. Finally, the catalyst was separated by filtration, and the products of the reaction mixture were analyzed by GC using dodecane as internal standard and confirmed with GC-MS.

#### Calculation of Conversions and Selectivities

For all the cases, the calibration was done using dodecane as the internal standard. The response factor of the analytes (RF_*i*_) was determined by injecting known quantities of each analyte *i* into the corresponding GC.
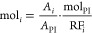
where A_*i*_ is the area of the analyte, A_PI_ is the area of the standard (dodecane), and mol_PI_ the exact molar amount of the standard added. Then, conversions and selectivities were calculated as follows:
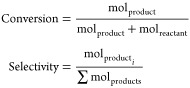


### 5 Measurement of *T*_bulk_ Using a Fiber-Optic Temperature Sensor

To determine the solution temperature (*T*_bulk_) during the catalysis, we used a novel type of sensor based on regenerated fiber Bragg gratting (RFBGs) that was developed by the PRL-ITEAM Research Institute of Universitat Politècnica de València.^50^ RFBG sensors measure variations in the wavelength of the light that is transmitted in response to a variation in temperature, making the temperature measurement highly precise (registering temperatures up to 1200 °C with an accuracy of 0.5 °C). More specifically, the RFBG sensor was inserted into the reactor through a thin quartz pod (see Figure S38 in SI section S13), and the temperature registered with the help of a LUNA interrogator (model Hyperion si155).

## Supporting Information Available

Experimental procedures, analytical data, and characterization data (PDF)
