# Supplementary material for: Magnetically Induced Catalytic Reduction of Biomass-Derived Oxygenated Compounds in Water
Source: ACS Catal. 2022 Jul 1;12(14):8462–75. doi: 10.1021/acscatal.2c01696 (PMC10388291; doi:10.1021/acscatal.2c01696)
Supplement: Supplementary file 1 — cs2c01696_si_001.pdf [file cs2c01696_si_001.pdf]

Supporting Information

Magnetically Induced Catalytic Reduction of Biomass-derived Oxygenated Compounds in Water

Christian Cerezo-Navarrete,[1] Irene Mustieles Marin,[2] Héctor García-Miquel,[3] Avelino Corma,[1] Bruno Chaudret[2] and Luis M. Martínez-Prieto[1,4,]*

Email: luismiguel.martinez@csic.es

[1] ITQ, Instituto de Tecnología Química, Universitat Politècnica de València (UPV), Av. de los Naranjos S/N, 46022, Valencia, Spain

[2] LPCNO, Laboratoire de Physique et Chimie des Nano-Objets, UMR5215 INSA-CNRS UPS, Institut des Sciences appliquées, 135, Avenue de Rangueil, F-31077 Toulouse, France.

[3] ITEAM Research Institute, Universitat Politécnica de Valencia, C/Camino de Vera s/n, E-46022 Valencia, Spain.

[4] Departamento de Química Inorgánica (University of Seville) – IIQ, Instituto de Investigaciones Químicas; Avda. Americo Vespucio 49, 41092 Seville, Spain.

Table of Content

S1. Raman 2
S2. STEM-HADDF and EDX 3
S3. XRD 5
S4. VSM 6
S5. SAR 6
S6. Kinetics 9
S7. TEM 18
S8. Dispersibility of FeCo@Ni NPs 20
S9. State-of-the-art catalysts for the reduction of HMF 21
S10. Stability Studies 22
S11. Benefits of carbon encapsulation 23
S12. Measurement of the magnetic field amplitudes generated by the AMF 23
S13. Catalytic set-up 27

S1. Raman

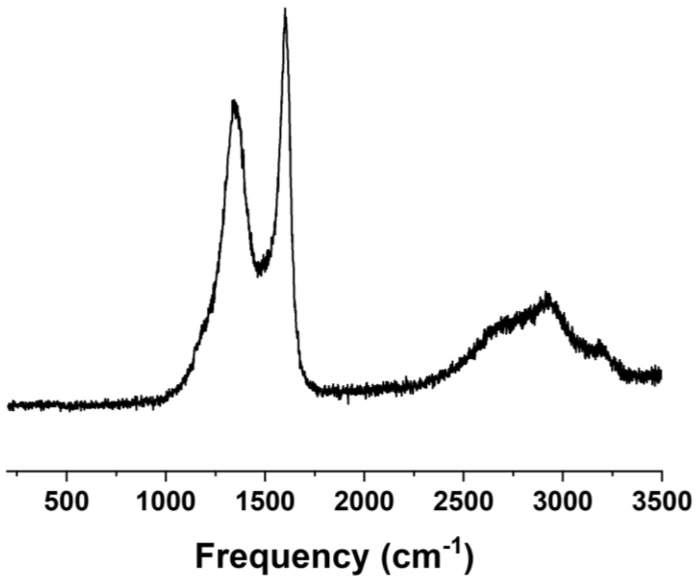

Figure S1. Raman spectra of **FeCo@Ni@C**.

S2. HRTEM, STEM-BF and EDX

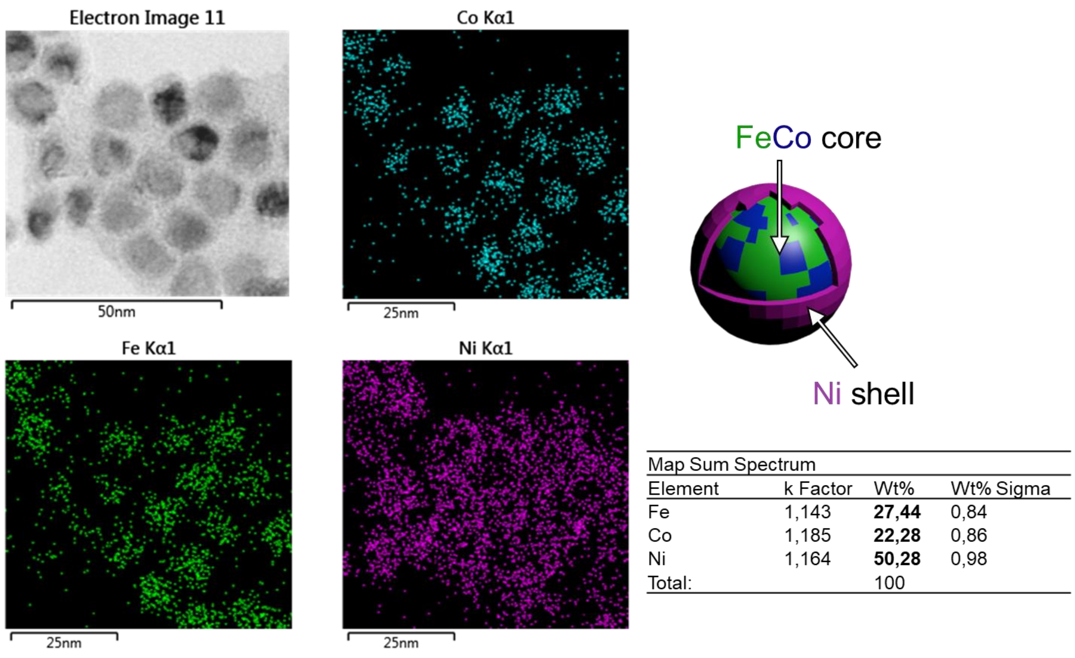

Map Sum Spectrum			
Element	k Factor	Wt%	Wt% Sigma
Fe	1,143	**27,44**	0,84
Co	1,185	**22,28**	0,86
Ni	1,164	**50,28**	0,98
Total:		100	

Figure S2. BF-STEM image, elemental mapping and relative composition profile of **FeCo@Ni** determined by EDX. Fe (green), Co (blue) and Ni (pink).

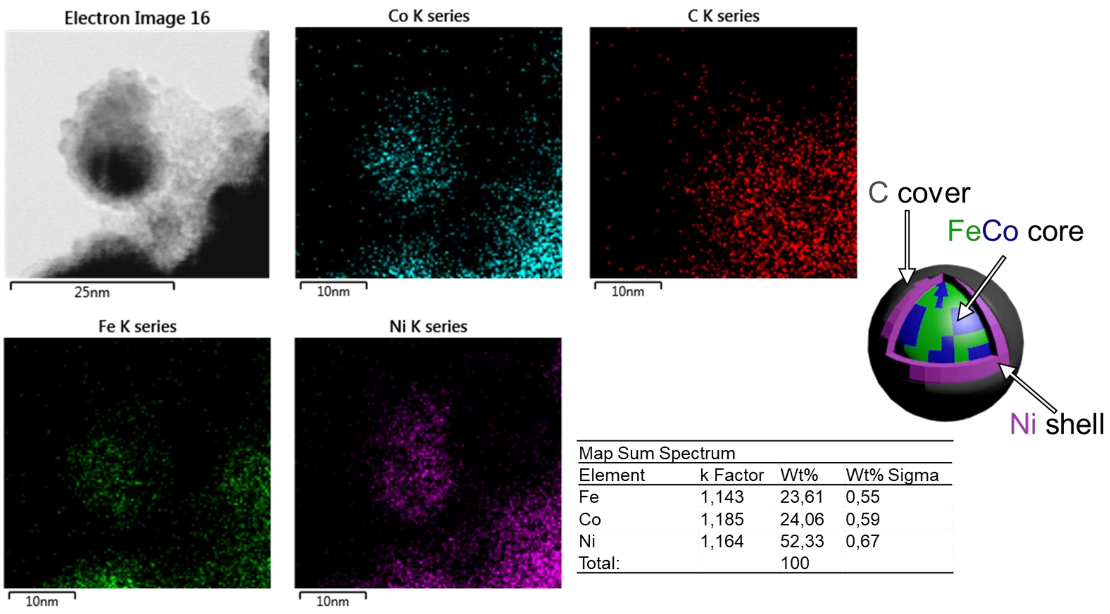

Map Sum Spectrum			
Element	k Factor	Wt%	Wt% Sigma
Fe	1,143	23,61	0,55
Co	1,185	24,06	0,59
Ni	1,164	52,33	0,67
Total:		100	

Figure S3. BF-STEM image, elemental mapping and relative composition profile of **FeCo@Ni@C** determined by EDX. Fe (green), Co (blue), Ni (pink) and C (red).

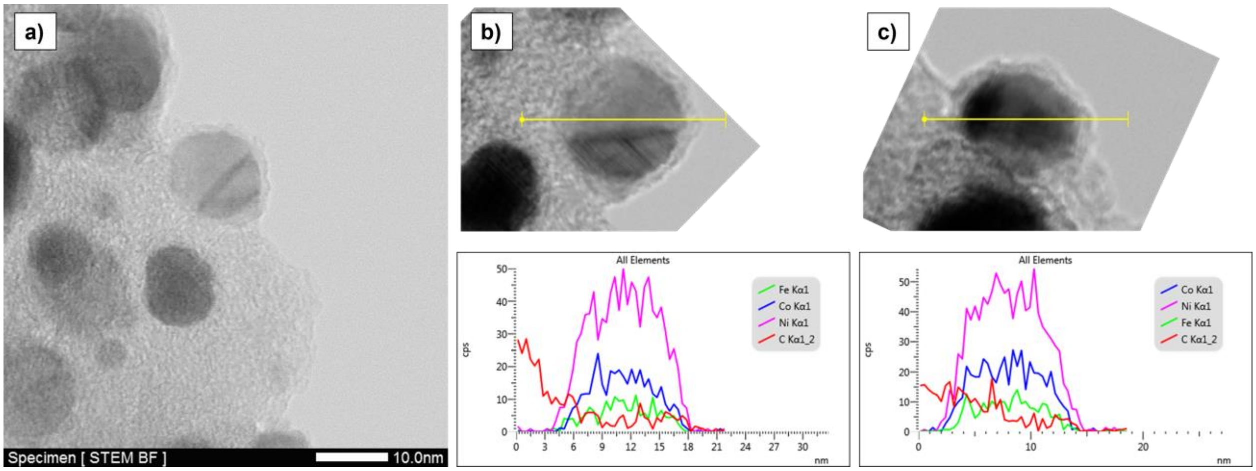

Figure S4. a) BF-STEM image and (b-c) relative composition profiles of **FeCoNi@C** after pyrolysis at 600 ºC determined by EDX.

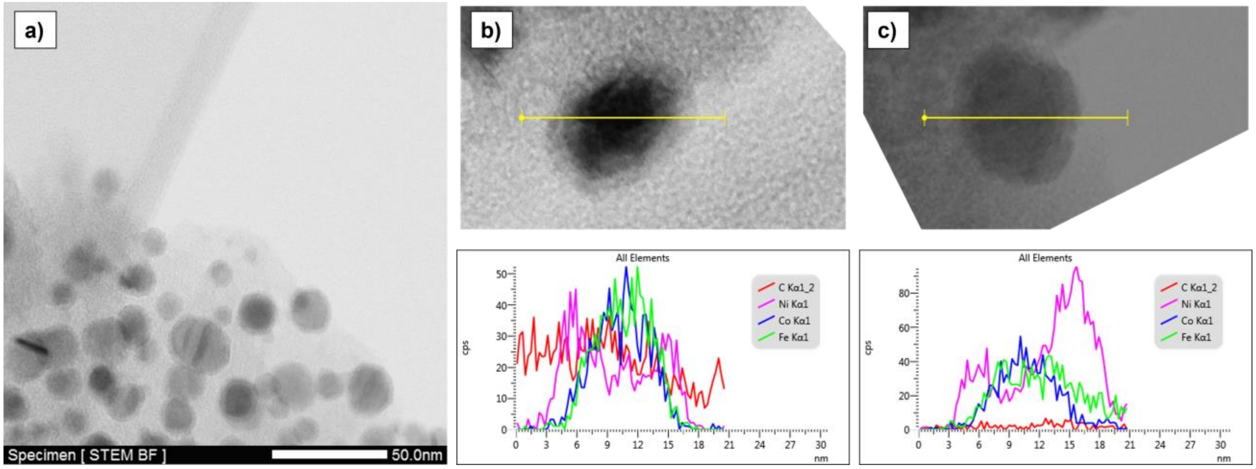

Figure S5. a) BF-STEM image and (b-c) relative composition profiles of **FeCoNi@C** after recycling experiment determined by EDX.

S3. XRD

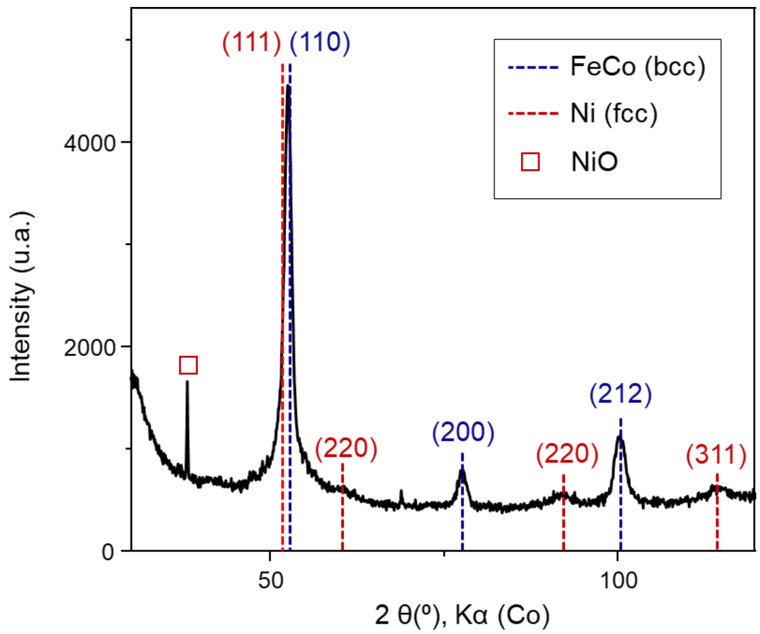

Figure S6. XRD diffractogram of **FeCo@Ni** NPs.

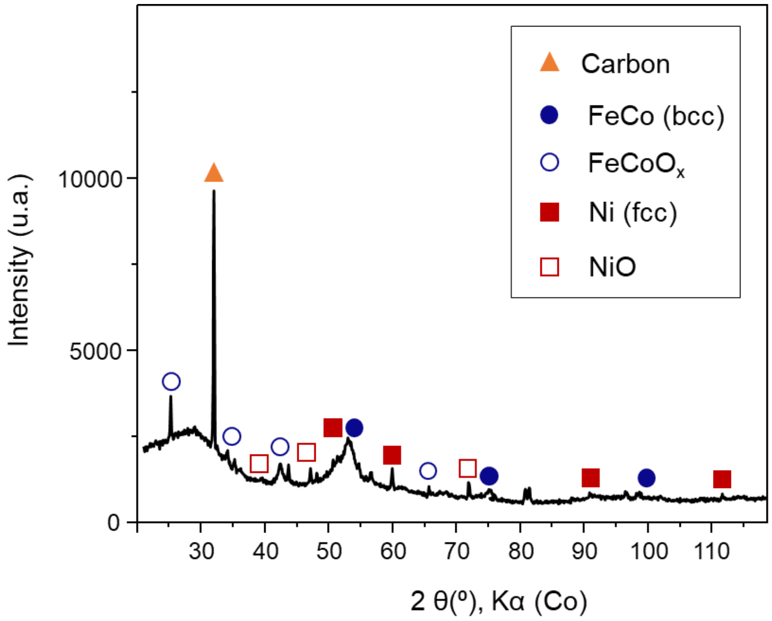

Figure S7. XRD diffractogram of **FeCo@Ni@C** NPs.

S4. VSM

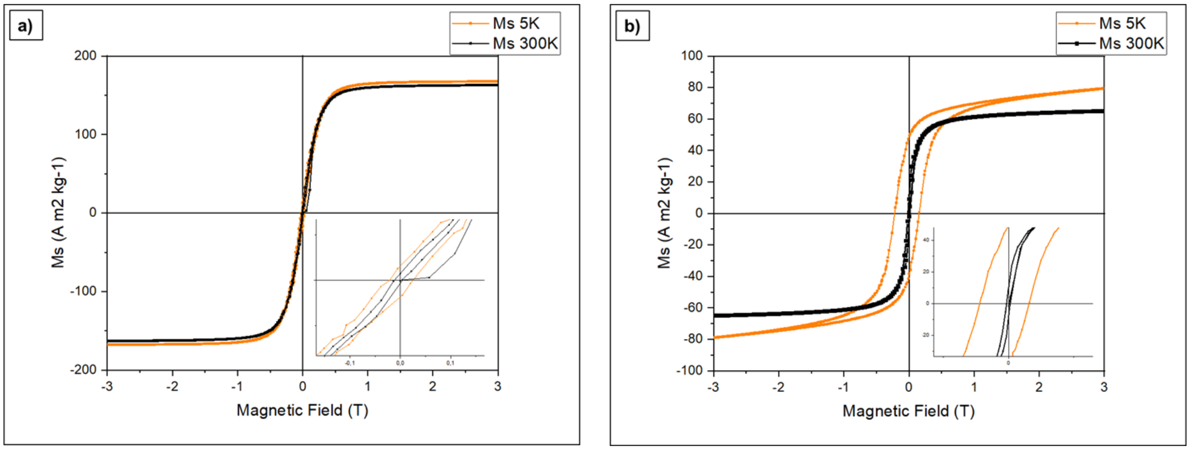

Figure S8. Hysteresis loops measured by VSM on **FeCo@Ni** (a) and **FeCo@Ni@C** (b) at 5 (orange) and 300 K (black) with zoomed region between -1 and 1 T.

S5. SAR

SAR has been measured by calorimetry. An air-tight tube containing about 10 mg of heating agents dispersed in 0.5 ml of mesitylene was filled under inert atmosphere. The tube was then placed in a calorimeter containing 2.5 ml of deionized water, the temperature of which was monitored during the experiment. The calorimeter was exposed to an alternative magnetic field for a time varying between 5 and 20s so that the temperature rise never exceeded 20°C. The temperature rise at the end of the magnetic field application was always measured after shaking the calorimeter to ensure the temperature homogeneity, which was measured by two probes (at the top and the bottom of the calorimeter). The temperature rise was determined after this process from the mean slope of the $\Delta T/\Delta t$ function. Then the raw SAR values were calculated using the expression:

$$\mathrm{SAR} = \frac{\sum_i C_{pi} m_i}{m_{met}} \times \frac{\Delta T}{\Delta t}$$

where C_{pi} and m_i are the specific heat capacity and the mass for each component respectively (Cp_{Fe} = 449 J.kg^{-1}.K^{-1}, Cp_{Co} = 421 J.kg^{-1}.K^{-1}, Cp_{Ni} = 440 J.kg^{-1}.K^{-1}, Cp = 1750 J.kg^{-1}.K^{-1}for mesitylene, Cp = 4186 J.kg^{-1}.K^{-1} for water and Cp = 720 J.kg^{-1}.K^{-1} for glass), and m_{met} is the mass of the pure metal in the sample.

The raw SAR values were corrected from the calorimeter losses, which were previously calibrated. For the calibration, a sample containing nanoparticles displaying moderate SAR was exposed for different time periods to an alternating magnetic field of 47 mT, 100 kHz. For each time, the SAR of the sample was measured. The SAR measured for an exposure time of 5s is considered as the “real” SAR (no losses). For longer exposure times, the difference between the measured SAR and the “real” SAR allows the determination of a corrective factor. The calibration curve is displayed below.

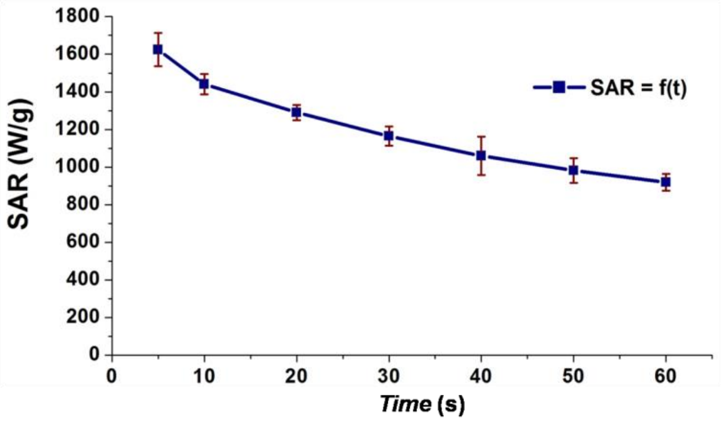

Exposure time (s)	Average SAR (W/g)	Corrective factor
5	1624	1,0
10	1440	1,11
20	1290	1,21
30	1165	1,28
40	1060	1,35
50	981	1,40
60	919	1,43

For each exposure time, the SAR was measured several times to ensure reproducibility. For the samples presented in this article, the measurement times were often comprised between 20 and 30s. Temperature monitoring has been done using a thermocouple disposed

in a quartz cannula disposed in the centre of the catalytic bed surrounded by the magnetic field inductor. Temperature range is 0-1300°C.

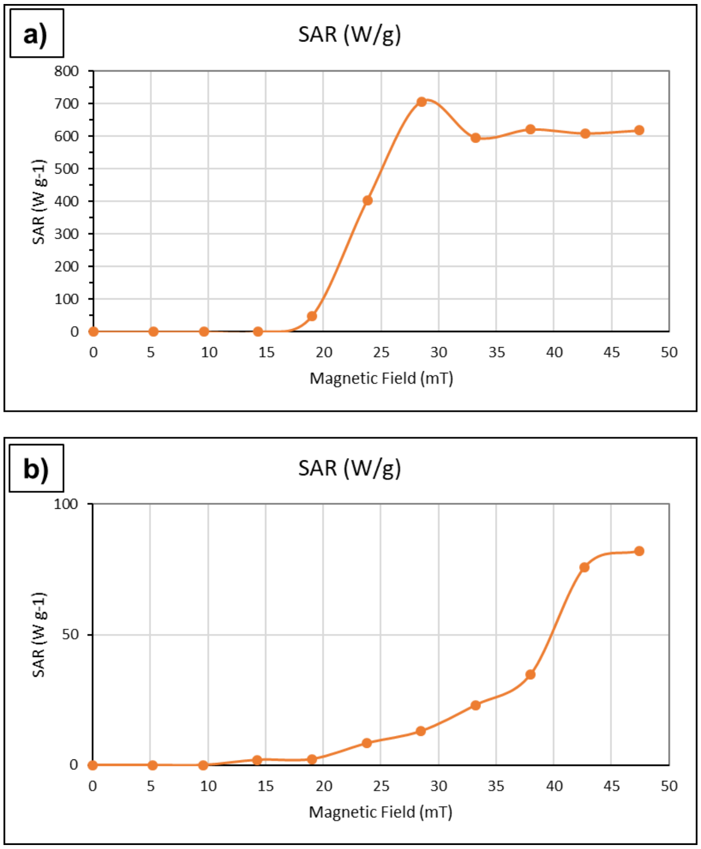

Figure S9. Specific absorption rate (SAR) of **FeCo@Ni** (a) and **FeCo@Ni@C** (b) measured by calorimetry in a 100 kHz alternating magnetic field.

S6. Kinetics

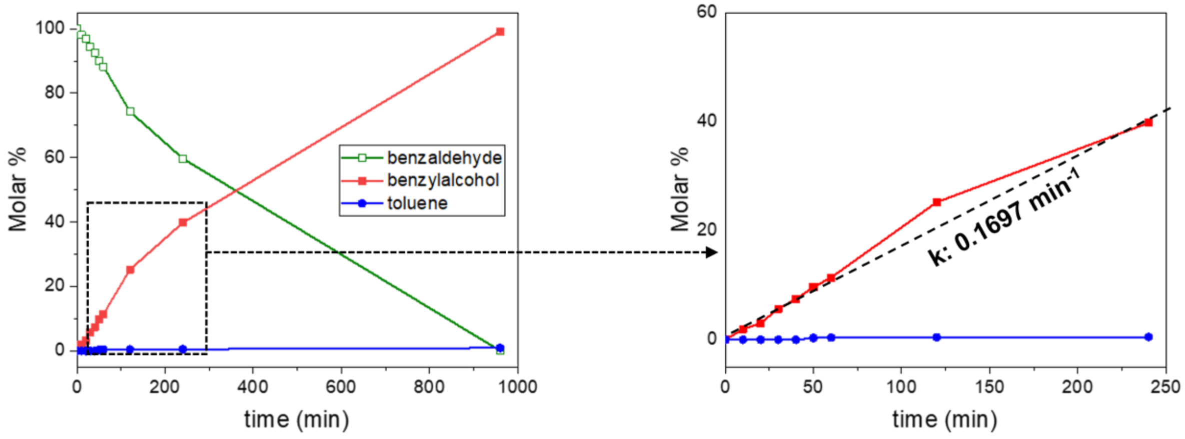

Figure S10. Catalytic transformation of benzaldehyde with **FeCo@Ni** NPs using conventional heating at 100 °C.

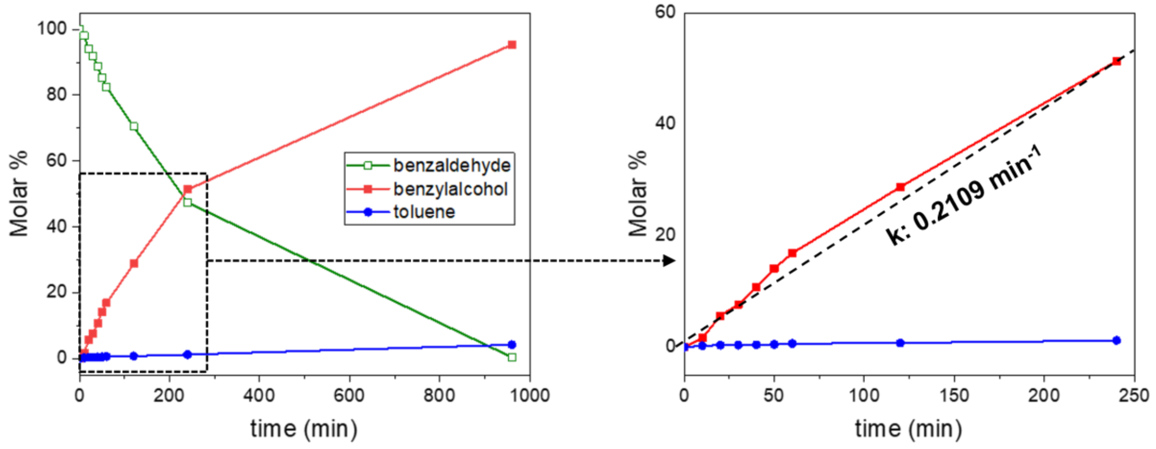

Figure S11. Catalytic transformation of benzaldehyde with **FeCo@Ni** NPs using conventional heating at 120 °C.

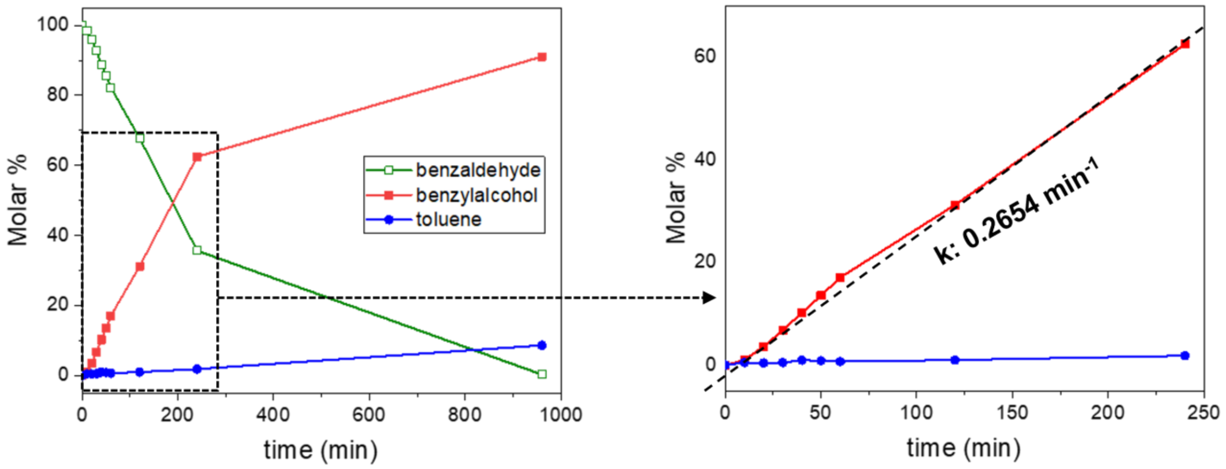

Figure S12. Catalytic transformation of benzaldehyde with **FeCo@Ni** NPs using conventional heating at 135 °C.

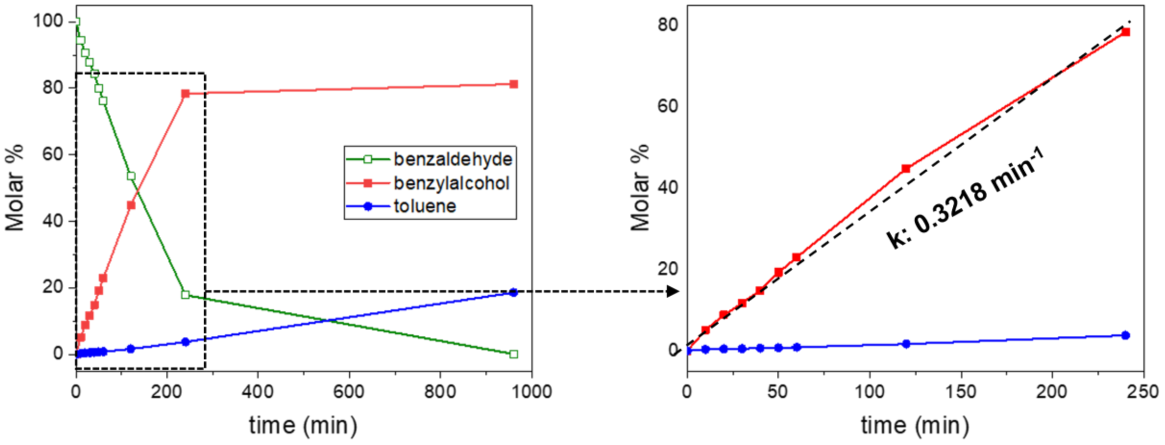

Figure S13. Catalytic transformation of benzaldehyde with **FeCo@Ni** NPs using conventional heating at 150 °C.

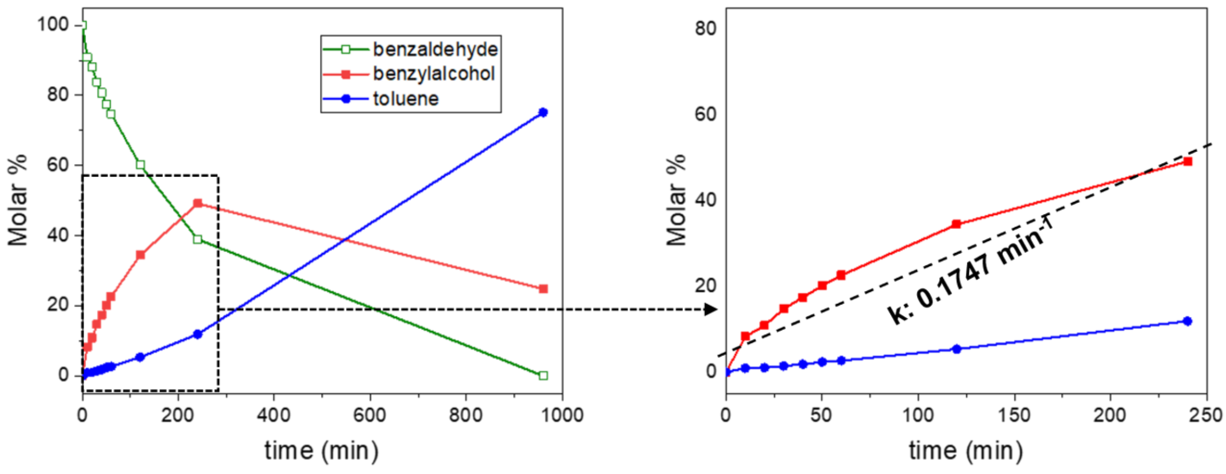

Figure S14. Catalytic transformation of benzaldehyde with **FeCo@Ni** NPs using conventional heating at 180 °C.

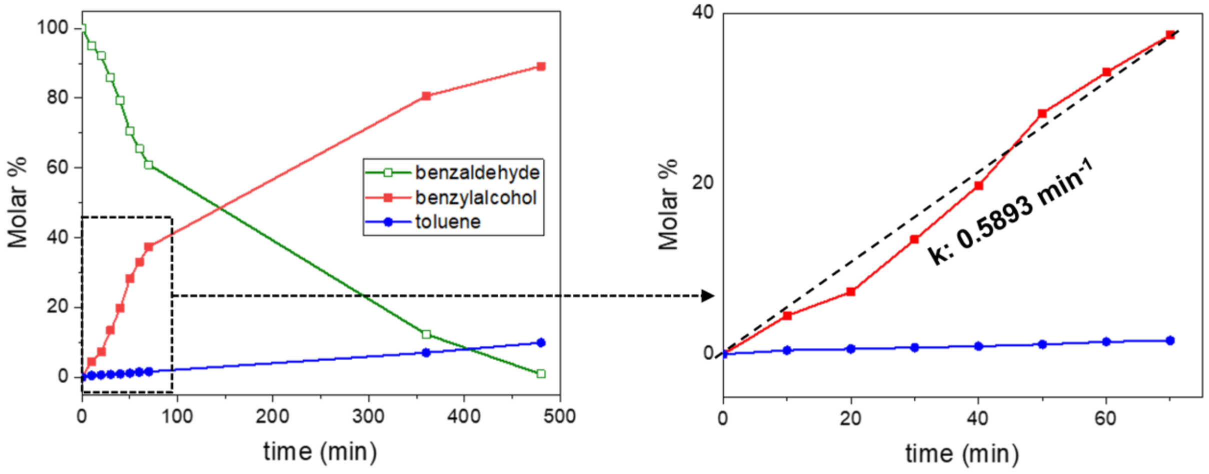

Figure S15. Catalytic transformation of benzaldehyde with **FeCo@Ni** NPs using magnetic heating at 35 mT (73 °C).

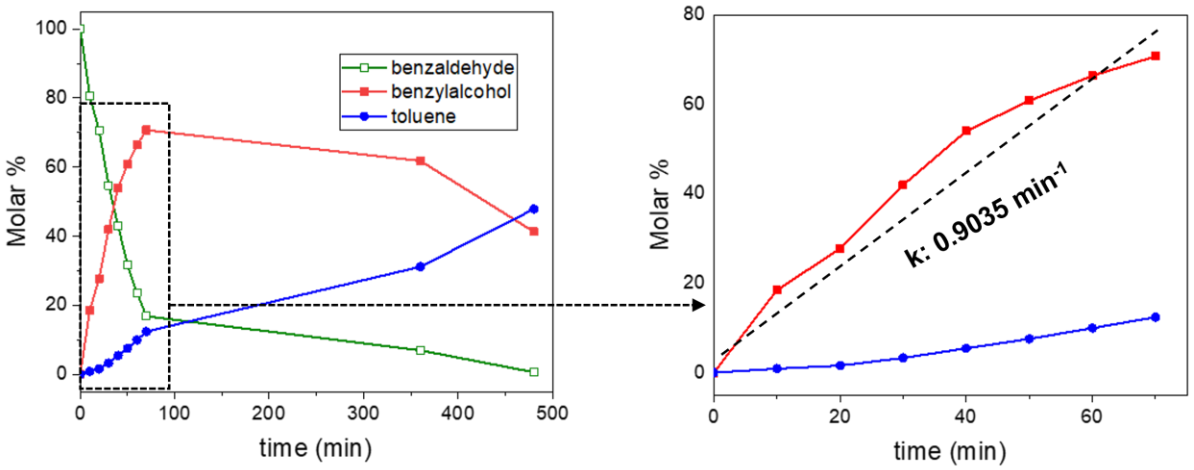

Figure S16. Catalytic transformation of benzaldehyde with **FeCo@Ni** NPs using magnetic heating at 60 mT (98 °C).

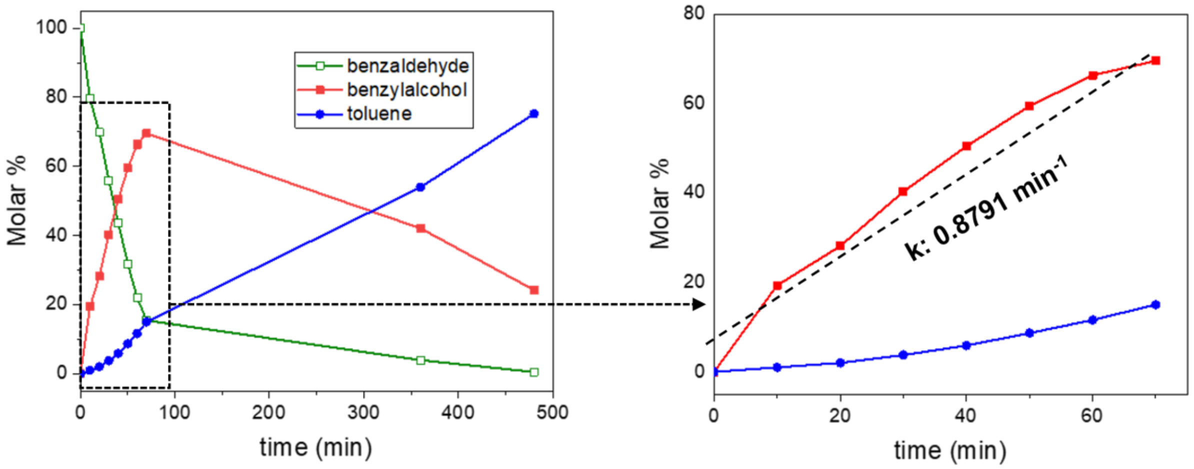

Figure S17. Catalytic transformation of benzaldehyde with **FeCo@Ni** NPs using magnetic heating at 83 mT (117 °C).

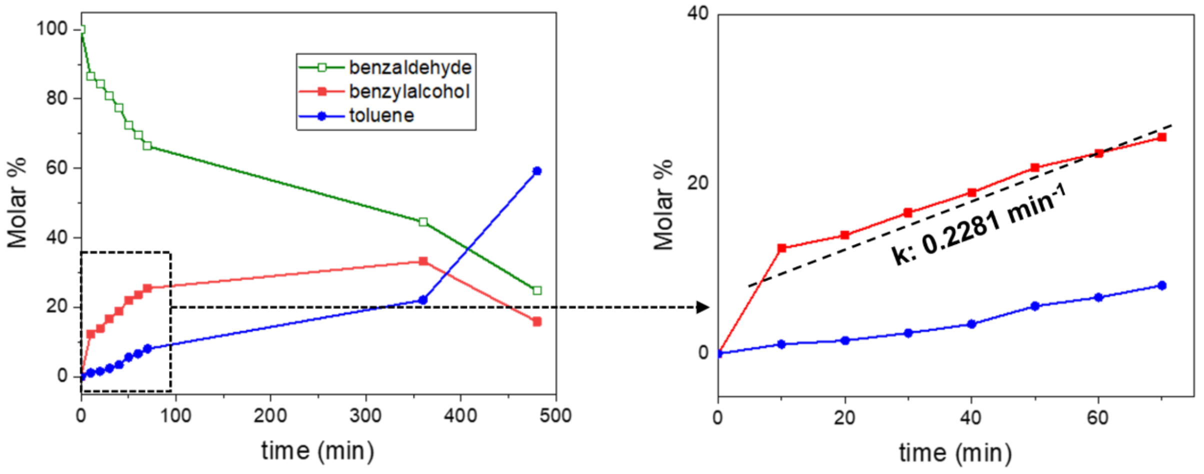

Figure S18. Catalytic transformation of benzaldehyde with **FeCo@Ni** NPs using magnetic heating at 100 mT (130 °C).

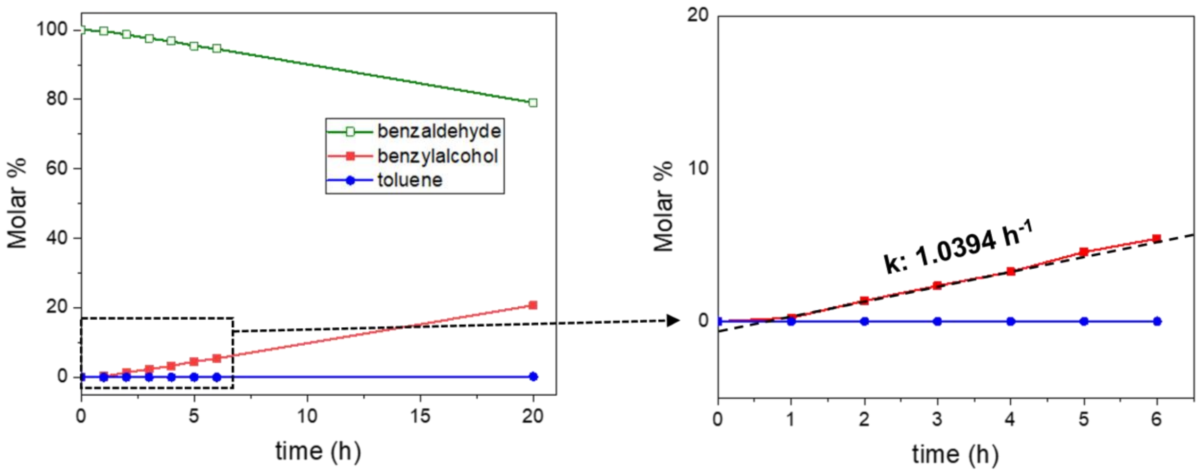

Figure S19. Catalytic transformation of benzaldehyde with carbon-coated **FeCo@Ni@C** using conventional heating at 75 °C.

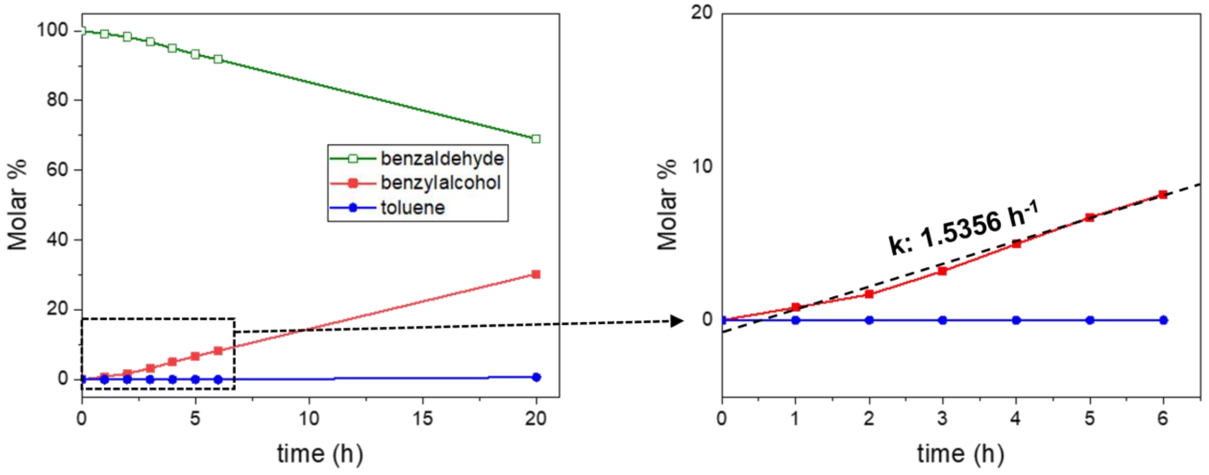

Figure S20. Catalytic transformation of benzaldehyde with carbon-coated **FeCo@Ni@C** using conventional heating at 90 °C.

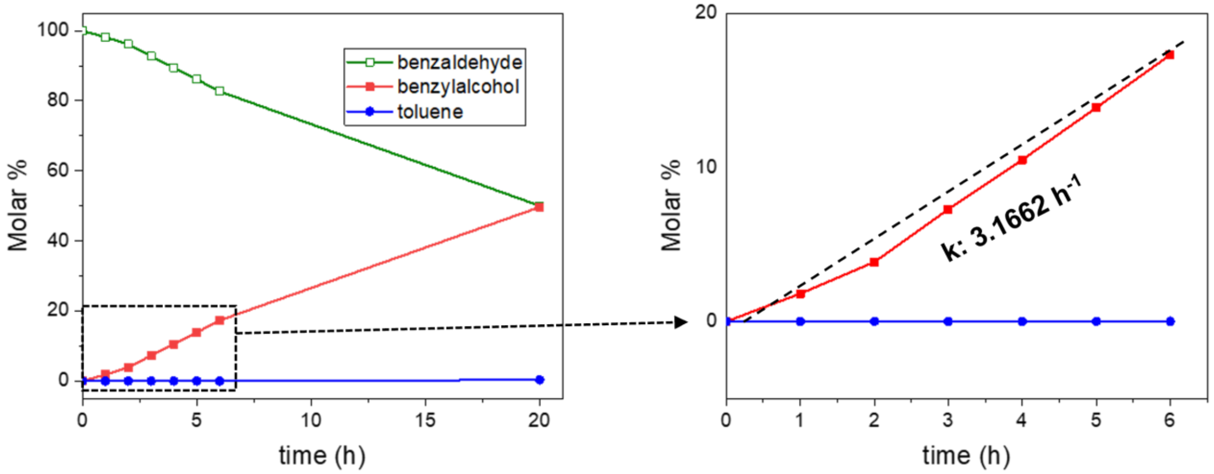

Figure S21. Catalytic transformation of benzaldehyde with carbon-coated **FeCo@Ni@C** using conventional heating at 110 °C.

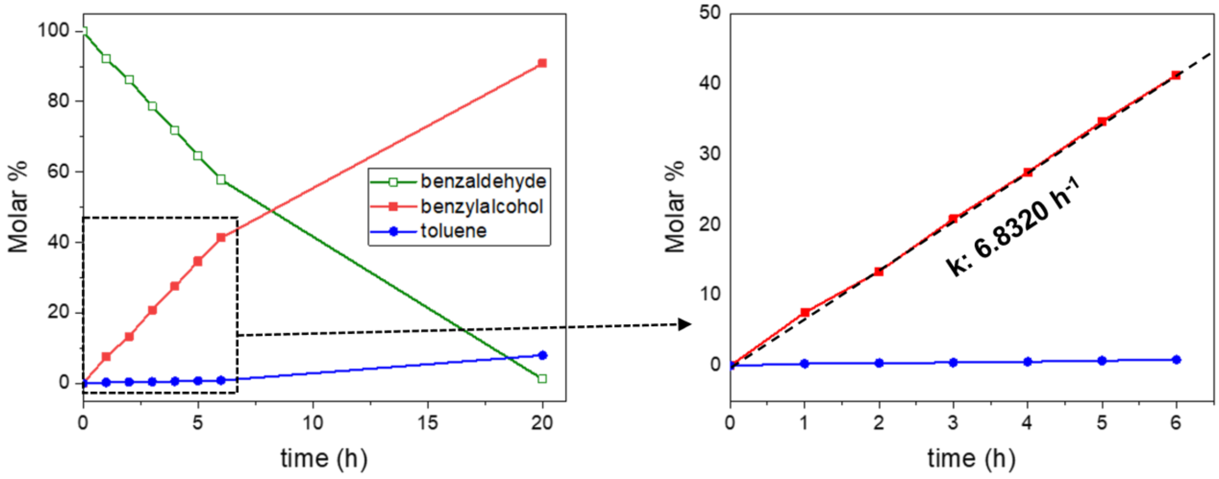

Figure S22. Catalytic transformation of benzaldehyde with carbon-coated **FeCo@Ni@C** using conventional heating at 154 °C.

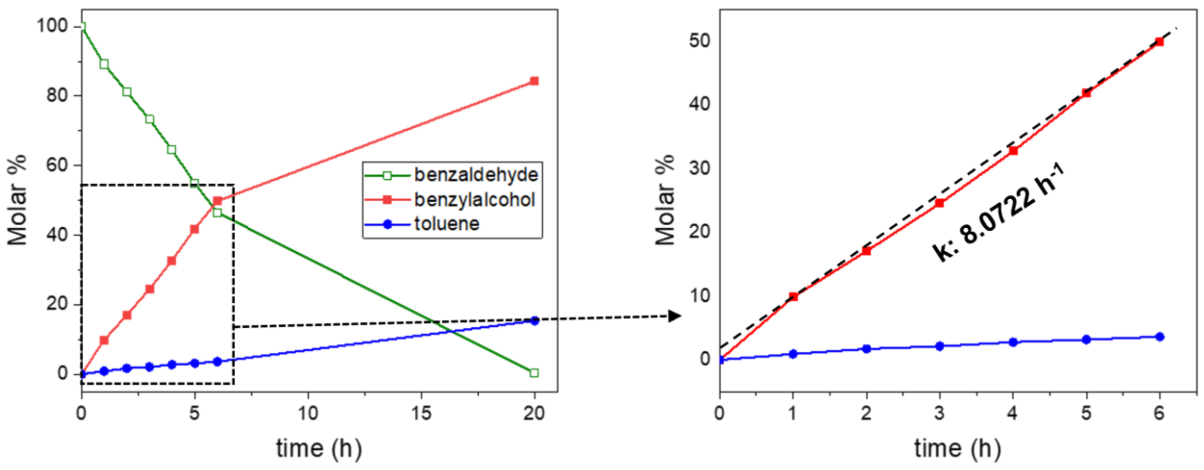

Figure S23. Catalytic transformation of benzaldehyde with carbon-coated **FeCo@Ni@C** using conventional heating at 180 °C.

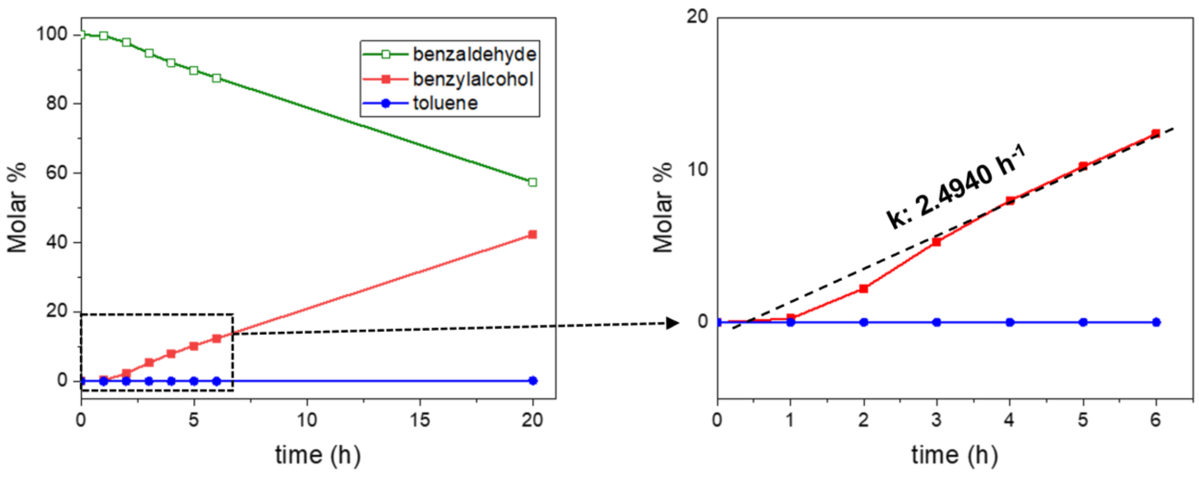

Figure S24. Catalytic transformation of benzaldehyde with carbon-coated **FeCo@Ni@C** using magnetic heating at 67 mT (58 °C).

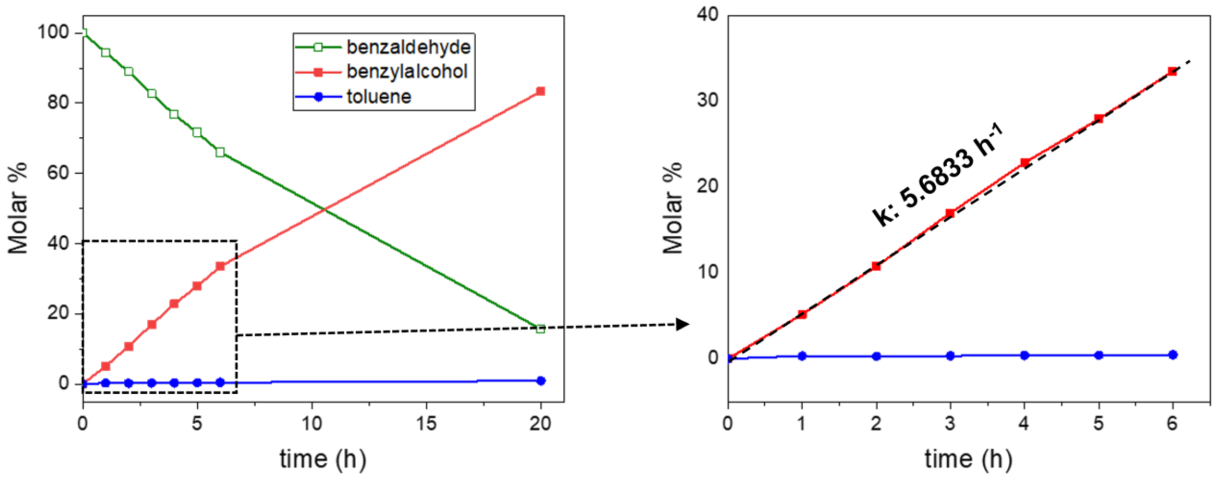

Figure S25. Catalytic transformation of benzaldehyde with carbon-coated **FeCo@Ni@C** using magnetic heating at 83 mT (75 °C).

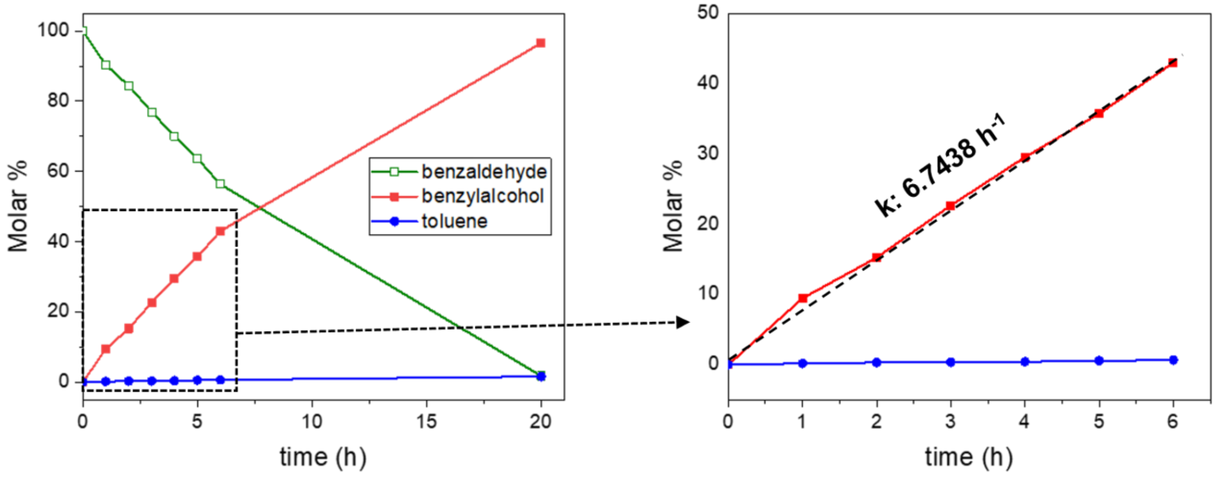

Figure S26. Catalytic transformation of benzaldehyde with carbon-coated **FeCo@Ni@C** using magnetic heating at 93 mT (90 °C).

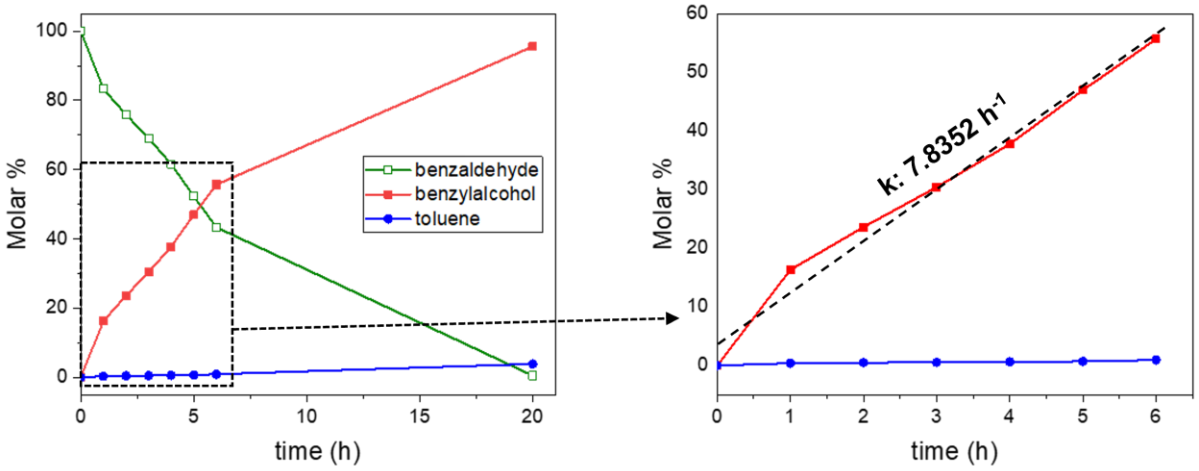

Figure S27. Catalytic transformation of benzaldehyde with carbon-coated **FeCo@Ni@C** using magnetic heating at 105 mT (110 °C).

S7. TEM

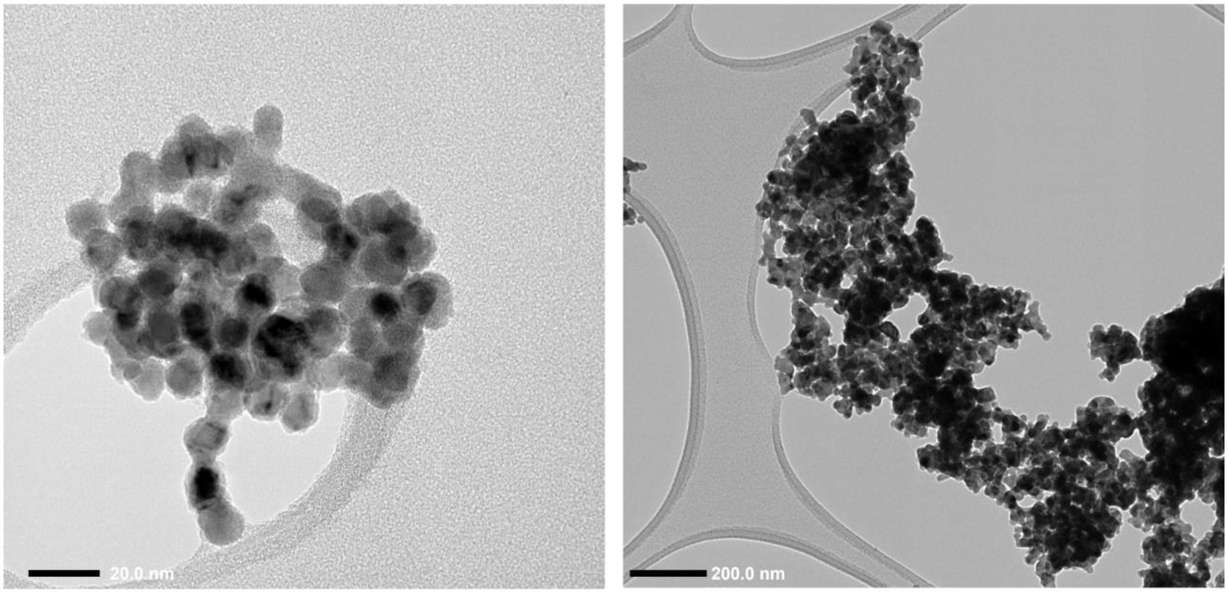

Figure S28. TEM image and size histogram of **FeCo@Ni NPs** after catalysis under conventional heating at 180 ºC.

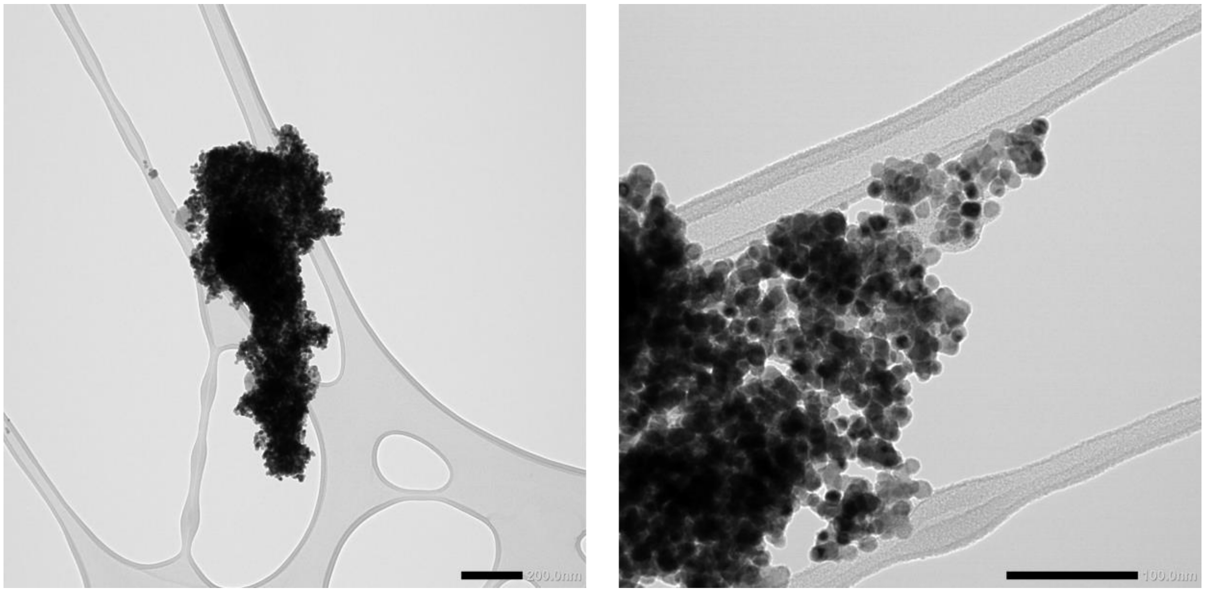

Figure S29. TEM image and size histogram of **FeCo@Ni NPs** after catalysis under magnetic heating at 100 mT.

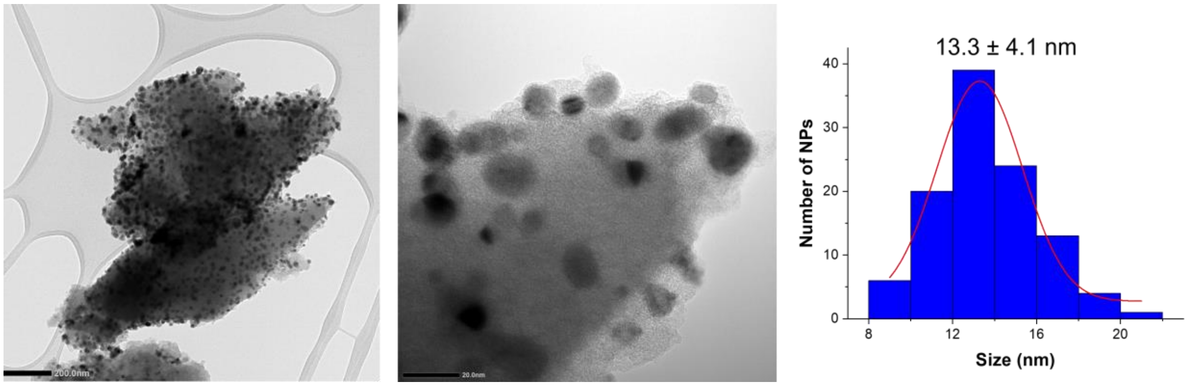

Figure S30. TEM image and size histogram of **FeCo@Ni@C** catalyst after recycling experiment in dioxane.

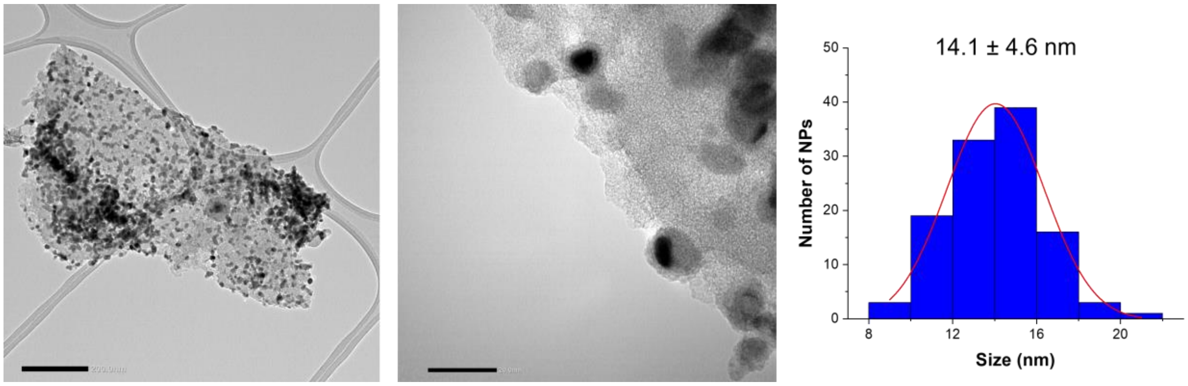

Figure S31. TEM image and size histogram of **FeCo@Ni@C** catalyst after recycling experiment in water.

S8. Dispersibility of FeCo@Ni NPs

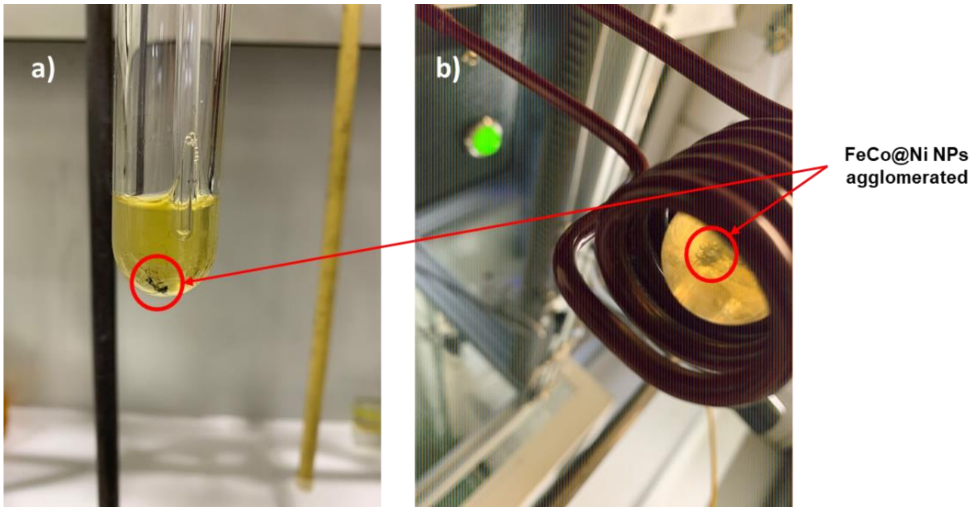

Figure S32. Dispersibility of **FeCo@Ni NPs** after sonication (a) and under the magnetic field (b) in water.

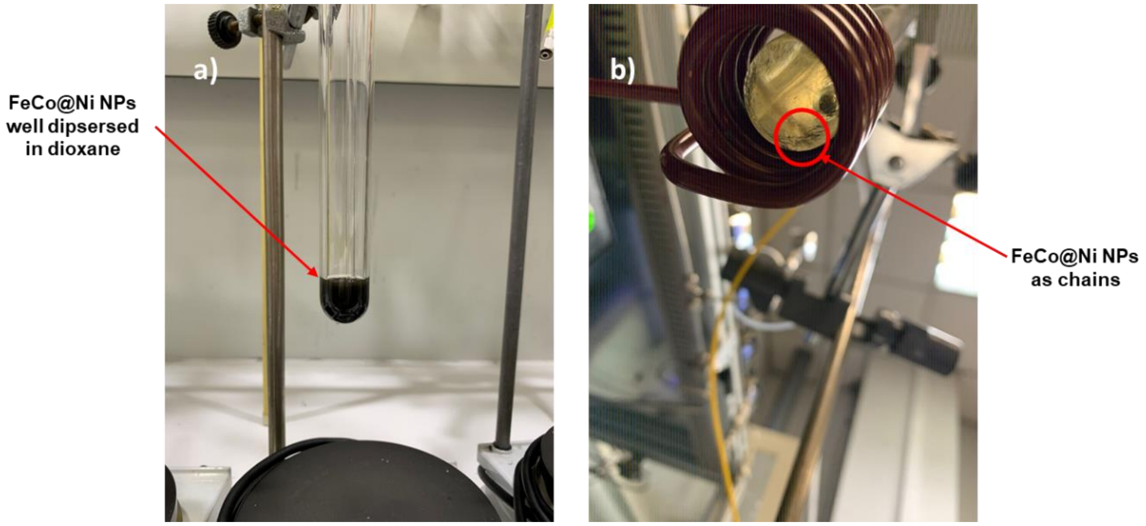

Figure S33. Dispersibility of **FeCo@Ni NPs** after sonication (a) and under the magnetic field (b) in dioxane.

S9. Table S1. State-of-the-art catalysts for the reduction of HMF.

Entry	Catalyst	Catalyst/HMF (mol %)	Solvent	Temp. (°C)	H_2 (bar)	Time (h)	Conversion (%)	Selectivity (%)			Reference
								BHMF	BHMTHF	DMF	
1	Pt/MCM-41 (1 wt%)	0.15	H_2O	35	8	2	>99	99	-	-	[1]
2	Pt/Y (1.3 wt%)	1	H_2O	80	20	4	>99	100	-	-	[2]
3	Ru/C (5 wt%)	5	THF	200	20	2	>99	-	-	94.8	[3]
4	Ru/MSN-Zr (5 wt%)	3	H_2O	25	5	4	98.1	92	5	-	[4]
5	Ru/$MnCo_2O_4$ (4 wt%)	0.1	methanol	100	82	4	>99	98	2	-	[5]
6[a]	Fe_2C/Ru (7 wt%)	0.1	mesitylene	165	3	15	>99	-	-	100	[6]
7	Raney Ni	72	1,4-dioxane	100	15	30	>99	-	96	2	[7]
8	Raney Ni	72	1,4-dioxane	180	15	15	>99	-	-	89	[7]
9	Ni/Al_2O_3 (47 wt%)	14	H_2O	80	20	6	>99	25	71	-	[8]
10	Ni/ZSM-5 (40 wt%)	34	THF	180	2.5	7	91	-	-	96	[9]
11	Ni-Ce/Al_2O_3 (10 Ni, 15 Ce wt%)	3.5	THF/water	140	50	6	>99	96	4	-	[10]
12	$Ni_{1.5}Co_1$ (45 wt%)	28	THF	100	10	1	99	93	-	-	[11]
13	NiCo/C (2 Ni, 20 Co wt%)	38	H_2O	210	15	24	99	-	-	61	[12]
14	NiZnAl (25 Ni, 29 Zn wt%)	11	1,4-dioxane	180	15	15	>99	-	-	94	[13]
15	Ni/WO_3 (5 wt%)	10	H_2O	180	10	6	>99	-	-	95	[14]
16	Ni/CNTs (10 wt%)	2.2	*n*-butanol	110	30	18	>99	76	-	5.5	[15]
17	Ni/CNTs (10 wt%)	2.2	*n*-butanol	200	30	3	>99	13	-	46	[15]
18[a]	$FeNi_3$@Ni (67 wt%)	12	Mesitylene	165	3	16	>99	-	-	100	[16]
19[a]	FeCo@Ni (35 wt%)	6	1,4-dioxane	75[b,c]	3	20	95	93	-	0	This work
20[a]	FeCo@Ni (35 wt%)	6	1,4-dioxane	106[b,d]	3	20	>99	-	-	100	
21[a]	FeCo@Ni@C (5 wt%)	8	1,4-dioxane	143[b,e]	3	20	>99	5	-	82	
22[a]	FeCo@Ni@C (5 wt%)	8	H_2O	95[a,e]	3	20	93	100	-	-	

[a] Magnetically induced catalysis. [b] T_{bulk} measured by a fiber-optic sensor. [b] 50 mT. [c] 83 mT. [d] 100 mT.

S10. Stability Studies

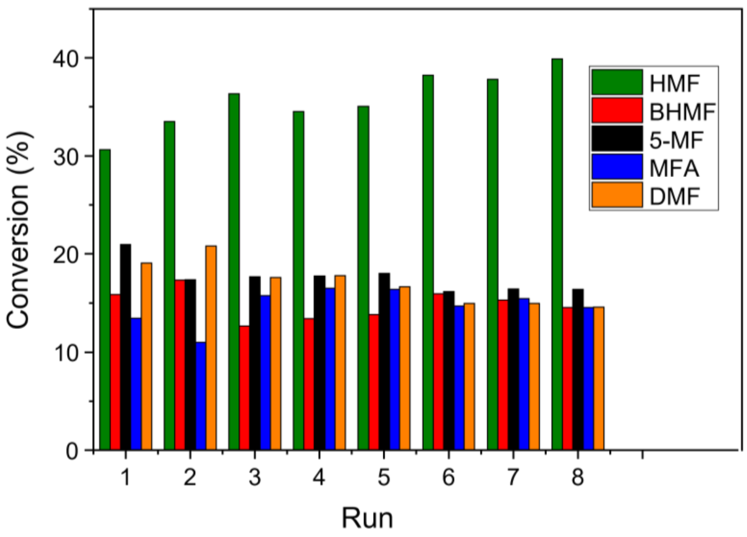

Figure S34. Recycling experiments using **FeCo@Ni@C** in dioxane.

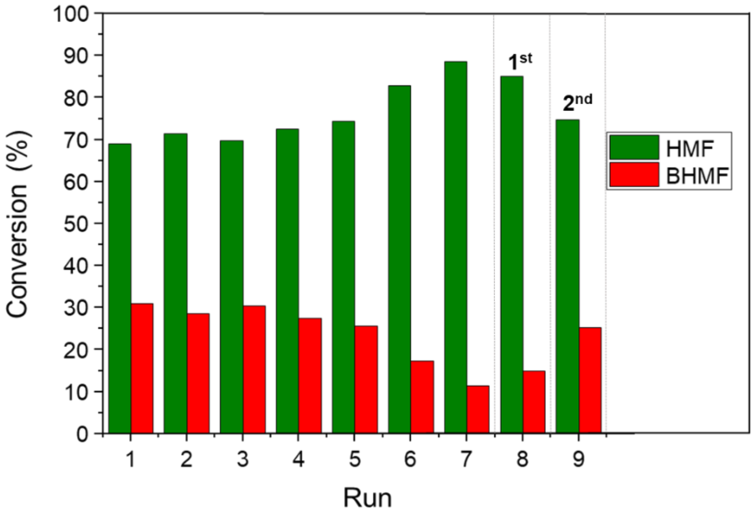

Figure S35. Recycling experiments using **FeCo@Ni@C** in water (regeneration treatments after the 7th run are separated by dashed lines).

S11. Benefits of carbon encapsulation

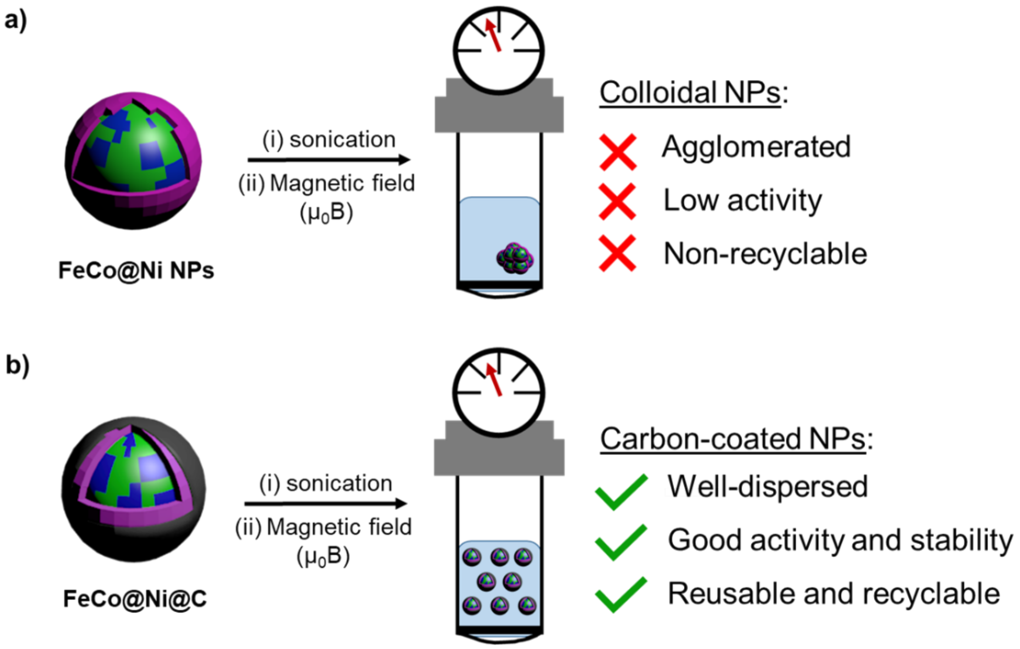

Scheme S1. Magnetically induced catalysis in solution using (a) colloidal and (b) carbon-encapsulated **FeCo@Ni** NPs.

S12. Measurement of the magnetic field amplitudes generated by the alternating magnetic field (AMF)

Given the operating mode of the AMF used in this work, which heat conductive or magnetic samples by induction, any sensor that presents metallic parts would be heated when trying to measure the magnetic field generated by the AFM, affecting the sensitivity of the sensor (or melting/deteriorating the sensor). In order to avoid these drawbacks, we decided to use a one loop pick-up coil made with a small diameter Cu wire of 90 μm to reduce heating by eddy currents as much as possible (Figure S34). When using a pick-up coil with one loop, even if it is heated, it would not affect the measurement since the voltage induced in the coil was measured with a high

impedance amplifier and the induced voltage was not affected. According to Faraday's law, a voltage proportional to the derivative of the magnetic flux over time will be induced in the pick-up coil. Therefore, to determine the magnetic induction amplitudes, we should integrate the voltage obtained in the pick-up coil. In the case that the generated magnetic flux is sinusoidal (as it is in our case), we would obtain a sinusoidal voltage in the pick-coil and the magnetic induction amplitude by means of the integral of the sinusoidal voltage induced in the pickup coil. Thus, the one loop coil was connected to an oscilloscope to measure the induced voltage. Then, according to Faraday's law we obtained a voltage induced in the coil which was proportional to the variation of the magnetic flux with time and to the number of loops in the pickup coil (N=1 in our case due to the high voltage induced in the one loop coil) :

$$e=-N\frac{d\phi}{dt}$$

Considering a constant magnetic induction, B, inside the pickup coil area and a sinusoidal magnetic flux:

$\phi=\oint B\,dS=B\,S \quad \text{with} \quad B=B_p\sin(\omega t)$

$\frac{d\emptyset}{dt}=S\,B_p\,\omega\,\cos(\omega\,t)$

$$e=-N\,S\,B_p\,\omega\,\cos(\omega\,t)=V_p\cos(\omega t)$$

The magnetic induction, B, determined through the voltage induced in the pick-up coil, is seen in the following equation, where B_P is the amplitude of the sinusoidal induction, V_P the amplitude of the induced voltage in the pick-up coil, N the number of loops in the pick-up coil and f the frequency of the magnetic induction:

$$B_p = \frac{V_p}{N * S * 2\pi * f}$$

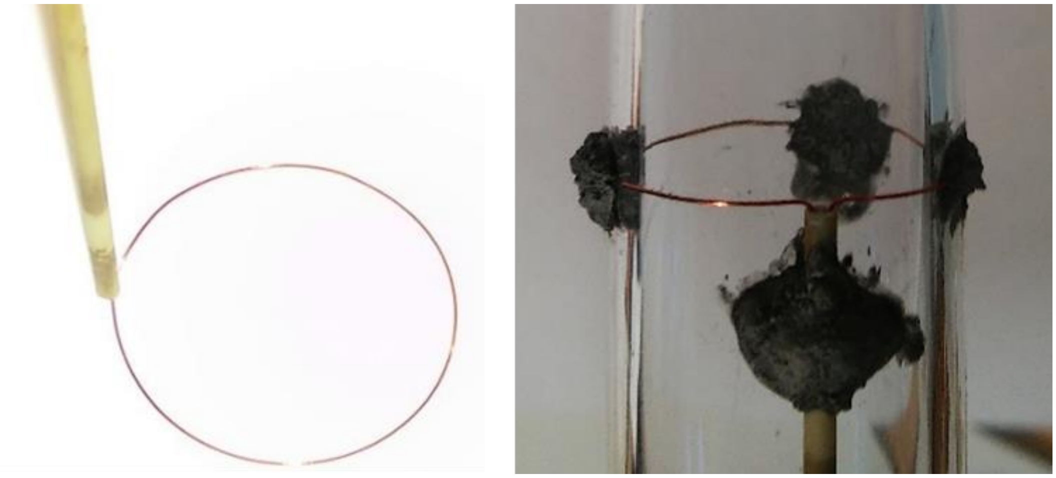

Figure S36. Pictures of the one loop pick-up coil with 90μm diameter wire with a diameter of 19mm and loop area of $2{,}836.10^{-4}$ m^2. The coil has been fixed to the quartz tube with glue that withstands 1200 ºC.

For the designed pick-up coil sensor and a frequency of 328.9 kHz (working frequency of the AMF with the 6-turn field-generating coil), the relationship of magnetic induction with the voltage in the coil is:

$$B_p = \frac{V_p}{N^* S_i^* 2\pi^* f} = \frac{V_p}{612.667} = 1.707 \cdot 10^{-3} * V_p \quad \text{[Teslas]}$$

Then, the magnetic induction in vacuum and the magnetic field (dividing the magnetic induction by μ_o) can be obtained as a function of the voltage induced in the pick-up coil:

$H_p = 1358{,}4 * V_p$ [A/m] $H_{RMS} = 960{,}5 * V_p$ [A/m]

B_p=1,707*V_p [mT] B_{RMS}=1,207*V_p [mT]

It is worth to mention that this calculation of the magnetic field is only valid if the magnetic flux is sinusoidal. If the magnetic field is not sinusoidal, it would be necessary to perform the integral of the signal obtained in the pick-up coil and divide it by the loop area and the vacuum permeability in order to get the magnetic field (this integral could be done with an analog integrator or done with a math program). In the measurements made in this AMF, the voltage obtained in the pick-up coil sensor was sinusoidal, so we could determine the induction and magnetic field proportional to the peak voltage obtained in the pick-up coil (Figure S35).

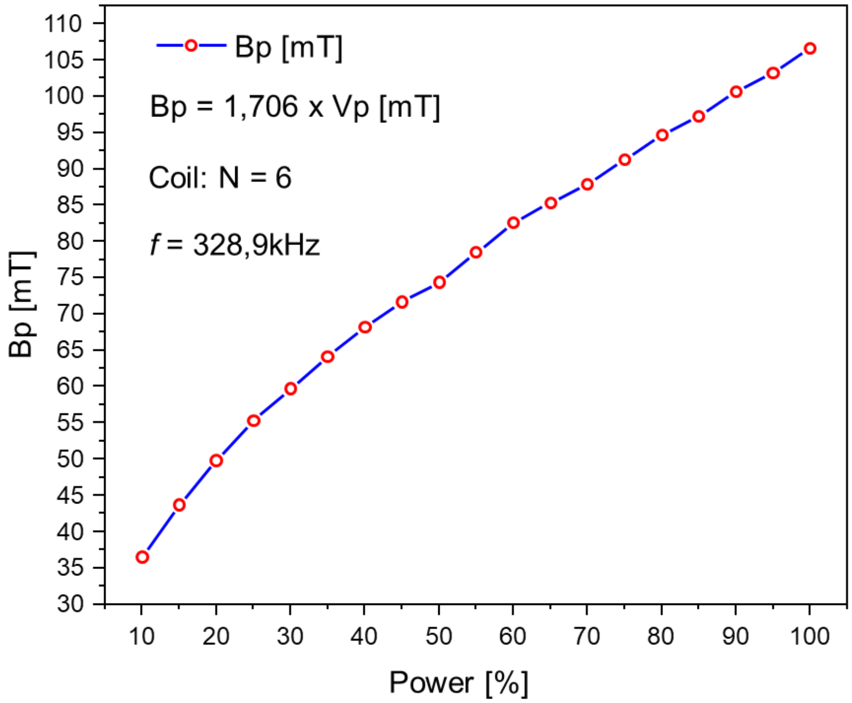

Figure S37: Magnetic induction generated (Bp) by the AMF at the frequency of 328.9 kHz. Bp values were determined from the voltage induced in the one loop pick-up coil sensor.

S13. Catalytic set-up

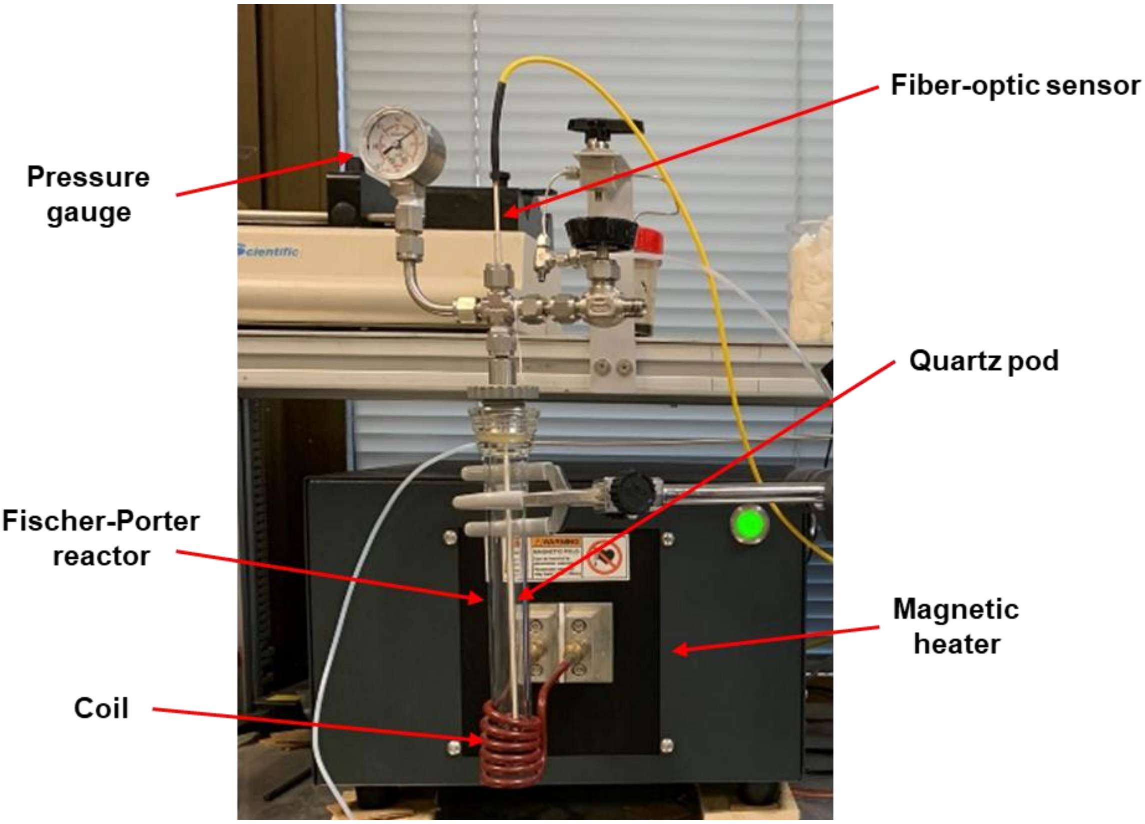

Figure S38. Picture of the catalytic set-up including the fiber-optic sensor.

References

[1] Chatterjee, M.; Ishizaka, T.; Kawanami, H. Selective hydrogenation of 5-hydroxymethylfurfural to 2,5-bis-(hydroxymethyl)furan using Pt/MCM-41 in an aqueous medium: a simple approach. *Green Chem.*, **2014**, *16*, 4734-4739.

[2] Chen, Q.; Li, T.; Zhou, Y.; Bi, Y.; Guo, S.; Liu, X.; Kang, H.; Wang, M.; Liu, L.; Xing, E.; Yang, D. Selective Hydrogenation of 5-Hydroxymethylfurfural via Zeolite Encapsulation to Avoid Further Hydrodehydroxylation. *Ind. Eng. Chem. Res.*, **2020**, *59*, 12004-12012.

[3] Hu, L.; Tang, X.; Xu, J.; Wu, X.; Lin, L.; Liu, S. Selective Transformation of 5-Hydroxymethylfurfural into the Liquid Fuel 2,5-Dimethylfuran over Carbon-Supported Ruthenium. *Ind. Eng. Chem. Res.,* **2014**, *53*, 3056-3064.

[4] Chen, J.; Lu, F.; Zhang, J.; Yu, W.; Wang, F.; Gao, J.; Xu, J. Immobilized Ru Clusters in Nanosized Mesoporous Zirconium Silica for the Aqueous Hydrogenation of Furan Derivatives at Room Temperature. *ChemCatChem* **2013**, *5*, 2822-2826.

[5] Mishra, D. K.; Lee, H. J.; Truong, C. C.; Kim, J.; Suh, Y.-W.; Baek, J.; Kim, Y. J. Ru/$MnCo_2O_4$ as a catalyst for tunable synthesis of 2,5-bis(hydroxymethyl)furan or 2,5-bis(hydroxymethyl)tetrahydrofuran from hydrogenation of 5-hydroxymethylfurfural. *Mol. Catal.,* **2020**, *484*, 110722.

[6] Asensio, J. M.; Miguel, A. B.; Fazzini, P.-F.; van Leeuwen, P. W. N. M.; Chaudret, B. Hydrodeoxygenation Using Magnetic Induction: High-Temperature Heterogeneous Catalysis in Solution. *Angew. Chem. Int. Ed.,* **2019**, *58*, 11306-11310.

[7] Kong, X.; Zhu, Y.; Zheng, H.; Dong, F.; Zhu, Y.; Li, Y.-W. Switchable synthesis of 2,5-dimethylfuran and 2,5-dihydroxymethyltetrahydrofuran from 5-hydroxymethylfurfural over Raney Ni catalyst. *RSC Adv.,* **2014**, *4*, 60467–60472.

[8] Perret, N.; Grigoropoulos, A.; Zanella, M.; Manning, T. D.; Claridge, J. B.; Rosseinsky, M. J. Catalytic Response and Stability of Nickel/Alumina for the Hydrogenation of 5-Hydroxymethylfurfural in Water. *ChemSusChem* **2016**, *9*, 521–531.

[9] Guo, D.; Liu, X.; Cheng, F.; Zhao, W.; Wen, S.; Xiang, Y.; Xu, Q.; Yu, N.; Yin, D. Selective hydrogenolysis of 5-hydroxymethylfurfural to produce biofuel 2,5-dimethylfuran over Ni/ZSM-5 catalysts. *Fuel* **2020**, *274*, 117853.

[10] Pomeroy, B.; Grilc, M.; Likozar, B. Process condition-based tuneable selective catalysis of hydroxymethylfurfural (HMF) hydrogenation reactions to aromatic, saturated cyclic and linear poly-functional alcohols over Ni-Ce/Al_2O_3. *GreenChem.*, **2021**, *23*, 7996–800

[11] Zhao, W.; Huang, Z.; Yang, L.; Liu, X.; Xie, H.; Liu, Z. Highly efficient syntheses of 2,5-bis(hydroxymethyl)furan and 2,5-dimethylfuran via the hydrogenation of biomass-derived 5-hydroxymethylfurfural over nickel-cobalt bimetallic catalyst. *Appl. Surf. Sci.,* **2022**, *577*, 151869.

[12] Yang, P.; Xia, Q.; Liu, X.; Wang, Y. Catalytic transfer hydrogenation/hydrogenolysis of 5-hydroxymethylfurfural to 2,5-demiethylfuran over Ni-Co/C catalyst. *Fuel* **2017**, *187*, 159-166.

[13] Kong, X.; Zhu, Y.; Zheng, H.; Zhu, Y.; Fang, Z. Inclusion of Zn into Metallic Ni Enables Selective and Effective Synthesis of 2,5-Dimethylfuran from Bioderived 5-Hydroxymethylfurfural. *ACS Sustainable Chem. Eng.,* **2017**, *5*, 11280−11289.

[14] Siddiqui, N.; Roy, A. S.; Goyal, R.; Khatun, R.; Pendem, C.; Chokkapu, A. N.; Bordoloi, A.; Bal, R. Hydrogenation of 5-hydroxymethylfurfural to 2,5-dimethylfuran over nickel supported tungsten oxide nanostructured catalyst. *Sustain. Energy Fuels* **2018**, *2*, 191−198.

[15] Yu, L.; He, L.; Chen, J.; Zheng, J.; Ye, L.; Lin, H.; Yuan, Y. Robust and recyclable nonprecious bimetallic nanoparticles on carbon nanotubes for the hydrogenation and hydrogenolysis of 5-hydroxymethylfurfural. *ChemCatChem* **2015**, *7*, 1701-1707.

[16] Mustieles Marin, I.; De Masi, D.; Lacroix, L.-M.; Fazzini, P.-F.; van Leeuwen, P. W. N. M.; Asensio, J. M.; Chaudret, B. Hydrodeoxygenation and hydrogenolysis of biomass-based materials using FeNi catalysts and magnetic induction. *Green Chem.,* **2021**, *23*, 2025-2036.
